# The Role of Antioxidants in the Connection Between Microbiota, Neuroinflammation and Epilepsy

**DOI:** 10.3390/biomedicines14030550

**Published:** 2026-02-27

**Authors:** Denise Maria Dardano, Maria Serra, Sara Ussia, Giovanna Ritorto, Carmen Altomare, Elisa Macrì, Rocco Mollace, Rocco Savino, Ernesto Palma, Rita Citraro, Carolina Muscoli, Maria Cristina Caroleo, Emilio Russo, Vincenzo Mollace, Roberta Macrì

**Affiliations:** 1 Pharmacology Laboratory Institute of Research for Food Safety and Health (IRC-FSH), Department of Health Sciences, University “Magna Graecia” of Catanzaro, 88100 Catanzaro, Italy; denisemaria.dardano@studenti.unicz.it (D.M.D.); carmen.altomare@studenti.unicz.it (C.A.); mollace@libero.it (V.M.); 2 Department of Experimental and Clinical Medicine, Laboratory of Mass Spectrometry and Proteomics, University “Magna Græcia” of Catanzaro, University Campus, Europa Avenue, 88100 Catanzaro, Italy; 3 Department of Experimental Medicine, University “Tor Vergata” of Rome, 00133 Rome, Italy; 4 Veterinary Pharmacology Laboratory, Institute of Research for Food Safety and Health (IRC-FSH), Department of Health Sciences, University “Magna Graecia” of Catanzaro, 88100 Catanzaro, Italy; 5 Department of Health Sciences, University “Magna Graecia” of Catanzaro, 88100 Catanzaro, Italy; citraro@unicz.it (R.C.);; 6 CRUISE Research Center, Science of Health Department, Magna Graecia University of Catanzaro, Viale Europa, 88100 Catanzaro, Italy; erusso@unicz.it; 7 Renato Dulbecco Institute, Lamezia Terme, 88046 Catanzaro, Italy

**Keywords:** microbiota dysfunction, epilepsy, gut–brain axis, glutathione, ketogenic diet, nutraceuticals, inflammation, oxidative stress

## Abstract

The gut microbiota’s (GM) regulation of inflammation and oxidative stress is supported by existing evidence, and its dysregulation relates to brain disease. Indeed, probiotics and prebiotics have been shown to improve cognitive function. This is associated with a stronger gut and blood–brain barrier and less gut inflammation. Oligofructose-enriched inulin alters the GM, reduces body fat, and lowers interleukin-6 (IL-6) in obese patients. Moreover, by increasing glutathione (GSH), the ketogenic diet (KD) prevents seizures and also benefits the intestinal short-chain fatty acid (SCFA) profile. Given the evidence on managing epileptic conditions, the aim of this review is to assess how changing the gut microbiota (GM) can be a therapeutic method for preventing neurodegenerative dysfunctions associated with epileptic seizure onset and progression, with a focus on innovative supplement strategies, including endogenous and exogenous antioxidants, nutrition, and new phyto-therapies. Indeed, though drugs are the main treatment for epilepsy, the KD and other supplements are increasingly being considered. These compounds affect neuronal excitability, neurotransmitter release, and neuroinflammation, thus providing an anticonvulsant effect. Specifically, the KD prevents seizures by increasing GSH levels, which represents a crucial endogenous antioxidant that plays a key role in counteracting neuroinflammation and gut microbiota dysfunction. Furthermore, due to their antioxidant and anti-inflammatory properties, plant extract derivatives may be new agents that could reduce neuroinflammation in seizures, affecting the gut–brain axis (GBA) through the intestinal microbiota. In conclusion, data suggest that further clinical studies are needed to explore how the GM impacts epilepsy, and how specific nutraceuticals might offer probiotic benefits. Thus, a combined effect of nutraceuticals and functional food might be appealing, potentially resulting in a more beneficial therapeutic outcome.

## 1. Introduction

### 1.1. Gut Bacterial Composition

Microbiota includes prokaryotes, single-celled organisms lacking nuclei that contain chromosomal deoxyribonucleic acid (DNA) and plasmids for horizontal gene transfer. The human microbiota consists of roughly 100 trillion (10^14^) microbes, ten times the number of eukaryotic cells in the body, with a total weight of 1.5–2 kg [1,2]. The microbiota’s microorganisms are categorized by kingdom (bacteria, eukaryotes, and archaebacteria), as well as phylum, class, order, family, genus, and species. Even though gut bacterial composition varies among individuals and is affected by numerous factors, the 160 non-pathogenic microorganisms in the human gut, 57 of which are common to everyone, belong to 5 microbial phyla: Firmicutes, Bacteroides, Actinobacteria, Proteobacteria, and Fusobacteria. The human GM includes many bacteria, mostly non-pathogenic, and a complex metagenome. It is an ecosystem with many niches, including the intestinal mucosa, our largest free surface at roughly 250–400 m^2^ [3,4]. The stomach’s acidity (low pH) limits the presence of bacteria. Lactobacilli, streptococci, and yeasts are the primary bacteria in the stomach’s mucous layer. In the duodenum, low microbial presence is caused by fast transit, secretions, and motor function due to the effect of propulsive motor activity that prevents stable colonization of the lumen. Species numbers grow gradually from the jejunum to the ileum (104 to 107), where Gram negatives and obligate anaerobes start to increase. The colon’s complex ecosystem is full of microbes (10^10^–10^12^ cells/gram), mainly anaerobes, especially in the cecum and colon, where substrates and environments support growth (low transit time, ready availability of nutrients, favorable pH, etc.) [5,6]. Hundreds of species in the colon are identifiable, while many others remain unknown. The majority of colon bacteria are anaerobes that do not produce spores. Pioneer bacteria influence the host’s epithelial cells’ gene expression after colonization. Adults typically harbor Firmicutes, mainly Gram-positive Clostridia, and Bacteroidetes, Gram-negative bacteria, making up 90% of the human GM [7]. In children, the gut contains various bacteria, but Actinobacteria, particularly Bifidobacteria, are most prevalent. Compared to children, the adult microbiota is more complex, with more bacteria and various microbial types. Children’s GM is affected by the mode of delivery (caesarean vs. natural) and initial nutrition (breastfeeding vs. formula milk). Indeed, the GM of children delivered by C-section differs from that of those delivered naturally due to a lack of exposure to vaginal microbiota [8]. Furthermore, breastfeeding is correlated with increased Bifidobacteria, which are highly protective. Bifidobacteria are crucial for newborns, easing gas and constipation, and are also essential during weaning because the gut changes as new foods are introduced. Furthermore, it has been observed that the start of weaning, i.e., the introduction of solid foods, leads to increased production of Bifidobacterium and a significant increase in 110 bacterial species in the intestine, mainly belonging to the Firmicutes phylum. This process can metabolize carbohydrates, synthesize vitamins, and degrade xenobiotics. At about 3 years old, a child’s gut bacteria composition resembles that of an adult. On the other hand, the microbiota becomes less varied when transitioning from adulthood to old age. The gut microbiome is increasingly recognized as a key regulator of metabolic, inflammatory, and neurocognitive processes [9].

### 1.2. GM and the Central Nervous System (CNS)

The connection between the microbiota and the CNS is established via the GBA, a two-way communication system that involves the CNS, the encephalon, the spinal cord, the autonomic nervous system (ANS), the enteric nervous system (ENS), and the hypothalamic–pituitary–adrenal (HPA) axis [10]. The autonomic nervous system, composed of sympathetic and parasympathetic parts, governs the movement of signals from the gut to the brain (afferent) and from the brain to the gut (efferent) [11]. The body’s primary stress response pathway is the HPA axis, which carefully controls the release of corticotropin-releasing factor (CRF), adrenocorticotropic hormone (ACTH), and GCs through its rhythmic activity. The rhythmic activity of the HPA axis is directly related to the body’s responses to stress, cognition, inflammation, and metabolism [12,13]. The CNS controls the gut environment through neuronal and endocrine pathways and impacting cells, such as immune cells, epithelial cells, and enteric neurons. The ENS is a highly specialized circuit that contains over 100 million neurons and can function autonomously from the CNS and spinal cord [14]. It also communicates bidirectionally with the CNS, using the sympathetic system via sensory and motor pathways through the prevertebral ganglia [15]. The ENS regulates gut functions, including movement, release, sensation, immunity, and hormone activity, by innervating visceral smooth muscle. The spinal cord and vagus nerve carry sensory signals to the brainstem and somatosensory areas, which are then modified by emotional and cognitive networks (ascending pathways) [16,17]. Simultaneously, the autonomic system’s efferent pathways (sympathetic and parasympathetic) link central emotional circuits to the ENS [18,19]. This leads to a complex interaction involving digestion, feelings, and the body’s physical state [20].

#### 1.2.1. GM Alterations and Neuroinflammation

Several studies reveal that gut bacteria affect brain activity, while brain structures influence microbiota, demonstrating a dynamic gut–brain axis. The research demonstrates that Bacteroides and Marvinbryantia, among other microbial groups, correlate with neural activity and white matter changes. Magnetic resonance imaging reveals a negative correlation between Bacteroides and brain activity, and Marvinbryantia is tied to lower fractional anisotropy in the cingulate gyrus. Further analysis using reverse magnetic resonance imaging (MRI) suggests that the brain impacts gut microbiota. The negative relationship between the right hippocampus’ volume and Intestinimonas suggests stress response region changes might alter the gut environment via the HPA axis or autonomic nervous system, thus changing microbial niches [21,22]. Likewise, the positive correlation between the left superior cerebellar peduncle volume and Ruminococcaceae UCG010 genus, a SCFA producer, suggests that cerebellar integrity, also through the regulation of gut motility, may promote conditions favorable to beneficial microbes [23,24]. Based on these findings, particular bacteria might limit brain activity, potentially by creating neurochemicals or affecting immune reactions. In line with the above, previous studies have indicated that bacteria of the genus Bacteroides are instrumental in the production of SCFAs and other substances that can cross the blood–brain barrier, affecting brain activity [25,26]. Acetate, propionate, butyrate, isobutyrate, valerate, isovalerate, and caproate, with their 1–6 carbon atom aliphatic tail, are short-chain fatty acids (SCFAs). These saturated fatty acids are mainly produced by the fermentation of dietary fibers by bacteria in the colon, including Bifidobacterium, Lactobacillus, Bacteroides, Ruminococcus, and Firmicutes [27,28].

**Figure 1:**
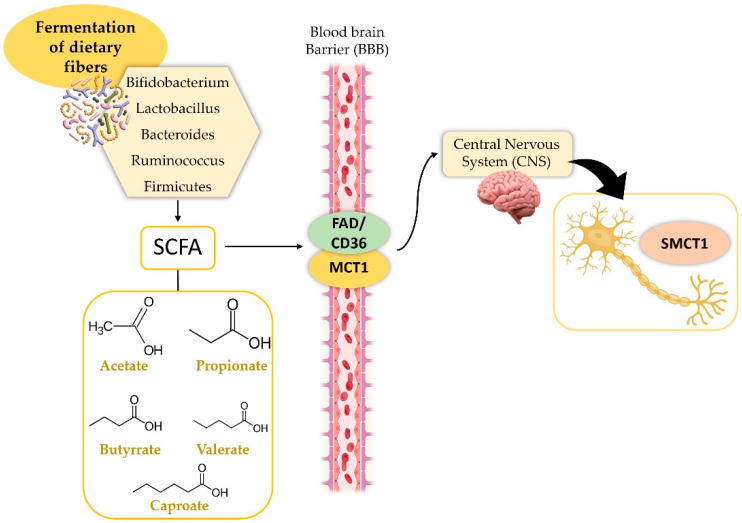
Gut-derived short-chain fatty acids (SCFAs) and neuronal transport mechanisms. SCFAs, including acetate, propionate, butyrate, valerate, and caproate, are generated through the fermentation of dietary fibers by gut bacteria such as Bifidobacterium, Lactobacillus, Bacteroides, Ruminococcus, and Firmicutes. These metabolites exert central effects via transporters like MCT1 and FAD/CD36, found at the blood–brain barrier, and Sodium-Coupled Monocarboxylate Transporter 1 (SMCT1), which is highly expressed in neurons. Abbreviations: Short-Chain Fatty Acids (SCFAs); Monocarboxylate Transporter 1 (MCT1); Fatty Acid Translocase Cluster of Differentiation 36 (FAD/CD36); Sodium-Coupled Monocarboxylate Transporter 1 (SMCT1); Central Nervous System (CNS).

SCFAs use Monocarboxylate Transporter 1 (MCT1), a monocarboxylate transporter, to cross the blood–brain barrier (BBB) [29], and Sodium-coupled Monocarboxylate Transporter 1 (SMCT1) (Solute Carrier Family 5 Member 8 (SLC5A8)), highly expressed in neurons [30]. Fatty Acid Translocase Cluster of Differentiation 36 (FAT/CD36) can mediate butyrate transport, as shown by in vitro models [30] (Figure 1).

Tight junctions prevent paracellular diffusion; therefore, transporters are the main way SCFAs enter the brain. The decrease in SCFAs, particularly butyrate, is connected with several neurological diseases, such as multiple sclerosis [31], stroke [32], traumatic brain injury [33], vascular dementia [34], septic encephalopathy [35], Alzheimer’s disease (AD) [36], and Parkinson’s disease [37]. One microbial metabolite that can cross the blood–brain barrier is tryptophan, which creates key molecules involved in gut–brain communication [38]. Since tryptophan cannot be synthesized endogenously, it must be obtained from the diet [39]. In the intestine, metabolism occurs through the kynurenine and serotonin (5-HT) pathways (with indoleamine 2,3-dioxygenase 1 (IDO1), tryptophan 2,3-dioxygenase (TDO), and tryptophan hydroxylase 1 (TPH1) involved), and the microbiota converts some into indole and its related forms [40,41]. Bile acids are also capable of crossing the blood–brain barrier via the Large Neutral Amino Acid Transporter 1 (LAT1) and act through the Farnesoid X receptor (FXR) and Takeda G-protein-coupled receptor 5 (TGR5). Bile acids and the microbiota engage in a bidirectional relationship that mutually modulates their composition [42]. The blood–brain barrier’s permeability to microbial bile acids, with known brain receptors, suggests these metabolites could aid the microbiota–gut–brain axis communication [43].

In mouse models of autism, problems with bile acid breakdown are associated with intestinal problems and behavioral changes, alongside a decrease in bile-metabolizing bacteria like Bifidobacterium and Blautia. Additionally, according to preclinical findings, bile acids may have a neuroprotective function in neuroinflammatory diseases such as Alzheimer’s, Parkinson’s, and Huntington’s, all of which are distinguished by neuroinflammation and microgliosis [44]. Considering the significant influence of the microbiota and its metabolites on brain function, interventions aimed at microbial composition could potentially change the risk or trajectory of neurological and psychiatric conditions, including depression, anxiety, neurodegenerative diseases, and developmental pathologies. Current therapeutic strategies aim to modulate the microbiota through diet, probiotics, prebiotics, and fecal microbiota transplantation (FMT). Diet is a primary way to alter gut microbiota, and it also affects the host metabolism [45,46]. Dietary habits significantly affect microbial balance, although the most substantial changes appear to occur at lower phylogenetic levels than the major phyla (Bacteroidetes and Firmicutes) [47]. Low-carb diets reduce Bifidobacterium spp., Roseburia spp., and Eubacterium rectale, thus reducing SCFA creation, especially butyrate [48]. The type and digestibility of carbohydrates also influence microbial composition. Resistant starch intake increases Ruminococcus bromii, Roseburia, and E. rectale, whereas a low-carbohydrate, high-protein diet can increase Oscillibacter valericigenes and reduce Roseburia and E. rectale [49]. Simultaneously, a diet rich in fiber supports the growth of Bifidobacterium, Ruminococcus, and the Lactobacillus–Enterococcus group [50]. According to the International Scientific Association for Probiotics and Prebiotics (ISAPP), probiotics are defined as “live microorganisms that, when administered in adequate amounts, confer a health benefit to the host” [51]. Probiotics typically add certain microbes to improve physiological processes and increase beneficial metabolites in the gut [51]. Preclinical studies have shown that supplementation with probiotics and prebiotics improves cognitive function in animal models by enhancing the integrity of the intestinal barrier and BBB, as well as modulating intestinal inflammation. Furthermore, both probiotics and prebiotics seem to help fix neurotransmitter systems, and beneficial outcomes have been seen in multiple neurodegenerative disease models [52,53,54].

#### 1.2.2. Reactive Oxygen Species (ROS) Effects on Microbiota–Gut–Brain Axis (MGBA) Alterations

Studies have shown a connection between GM issues and neurodegenerative and neurological diseases. Oxidative stress and neuroinflammation, though distinct, are connected and mutually influence each other.

An imbalance in ROS and free radicals, resulting in oxidative stress, has negative in vivo effects [55]. Furthermore, recent studies indicate that aging and various diseases are accelerated by increased superoxide anion (O_2_^−^), resulting from excessive ROS and free radical production. A surplus of ROS can be harmful, causing mitochondrial failure, protein damage, and lipid membrane destruction, potentially leading to cell death [55].

Particularly, the role of the microbiota in the management of brain alterations, including epilepsy, is caused by mitochondrial dysfunction, excess of ROS, and neuroinflammation [56]. O_2_^−^ is a major byproduct of mitochondrial metabolism, especially in oxidative phosphorylation and the TCA cycle, mainly at the level of respiratory chain complexes I (CI) and III (CIII). Thus, in mitochondria, peroxiredoxin 3 (Prdx3) and Thioredoxin reductase 2 (Trx2) frequently act as antioxidants. Physiological mtROS equilibrium is tightly maintained by the balance between free radical production and scavenging. However, once pathological conditions such as epilepsy occur, a surplus of ROS is produced, mainly in brain mitochondria, resulting in oxidative damage [55].

### 1.3. Microbiota and Epilepsy

#### 1.3.1. Pathophysiological Basis of Epilepsy

Epilepsy, a chronic neurological condition, involves repeated seizures. Drug-resistant epilepsy (DRE) develops in about a third of patients, despite the availability of anti-seizure medications (ASMs), which is a major clinical problem.

Epilepsy can be categorized into three main types: genetic generalized epilepsy (GGE), focal epilepsy, and epileptic encephalopathy (EE) [57]. Depending on the cause, epilepsy is usually classified into four groups: idiopathic, symptomatic, provoked, and cryptogenic; idiopathic and symptomatic are pure epilepsies [58]. Differences between various epilepsy types depend on things like seizures, electroencephalogram (EEG) patterns, age, and how the disease progresses. Generalized seizures impacting both brain hemispheres are a feature of GGE syndromes. Conditions such as juvenile myoclonic epilepsy (JME) and childhood absence epilepsy (CAE), which frequently appear in childhood or adolescence, frequently occur alongside normal development and intelligence. Unlike generalized seizures, focal seizures begin in one brain hemisphere. Examples include temporal lobe epilepsy, autosomal dominant nocturnal frontal lobe epilepsy (ADNFLE), and autosomal dominant epilepsy with auditory features. Severe, early-onset disorders known as epileptic encephalopathies (EEs) are characterized by uncontrollable seizures, developmental problems, regression, and persistent epileptic activity, frequently resulting in a negative outcome [59,60]. Studies on epilepsy genetics have shown new findings about somatic mosaicism causing focal epileptic lesions, repeat expansions, and familial epilepsy with myoclonus, and the increasing role of the non-coding genome in neurodevelopmental disorders.

Genetic studies identified specific mutations associated with GGE, particularly in genes coding for Gamma-aminobutyric acid (GABA)-A receptor subunits, such as Gamma-Aminobutyric Acid Type A Receptor Gamma 2 Subunit (GABRG2) and Gamma-Aminobutyric Acid Type A Receptor Alpha 1 Subunit (GABRA1) [61,62,63]. These mutations affect receptor affinity for GABA, transitions between active and inactive states, and membrane expression, resulting in neuronal hyperexcitability and a lower threshold for epileptic seizures [64]. Variants in GABRA1 are associated with JME but also with developmental and epileptic encephalopathies, characterized by early onset, pharmacoresistance, and psychomotor impairment [65]. Mutations in the Solute Carrier Family 2 Member 1 (SLC2A1) gene, which encodes the glucose transporter type 1, are present in 1% of patients with CAE, particularly in cases with very early onset, and may cause severe developmental encephalopathy or exercise-induced paroxysmal dyskinesia without cognitive impairment or epileptic seizures [66]. Genetic epilepsies encompass a broad spectrum of GGE syndromes, each characterized by specific seizure types, age of onset, and distinctive electroencephalographic findings. Other neurological diseases can cause provoked epilepsy, which is a secondary epilepsy [67].

Aging and genetics can cause increased brain electrical discharge in idiopathic seizures. Secondary, or symptomatic, seizures can stem from ailments, metabolic issues, and brain injuries such as focal lesions, abscesses, tumors, vascular malformations, and cerebrovascular accidents [67].

Additionally, the primary epigenetic mechanisms, including DNA methylation, histone post-translational modifications, and non-coding RNA expression/activity, are altered in both experimental and human forms of epilepsy [68]. In a study on temporal lobe epilepsy, the expression of DNMT1 and DNMT3A in patients with the condition versus healthy subjects was investigated. In particular, DNMT1 maintains methylation patterns, while DNMT3 drives new methylation. It was suggested by the data that both DNMT types are more common in individuals with temporal lobe epilepsy, a finding that points to their involvement in the condition’s pathogenesis [69].

A recent study analyzed the hippocampus of rats with chronic epilepsy for overall DNA methylation, revealing increased methylation in the epilepsy group compared to controls. A ketogenic diet was administered to the group, resulting in a decrease in seizure frequency and a modification of DNA methylation patterns [70].

Another study on the rodent hippocampus revealed distinct methylation patterns in animals with status epilepticus compared to those with epileptic tolerance. Preconditioning with chemical or electrical stimuli was performed on the latter group of mice before inducing status epilepticus; therefore, they exhibited tolerance to status epilepticus [71].

Evidence from animal epilepsy models suggests that histone modifications alter chromatin after seizures. Particularly, an increase in HDAC2 expression has been observed in tissue samples from temporal lobe epilepsy patients and in animal subjects undergoing status epilepticus, differing from control groups. Neurodevelopment involves the activity of HDAC2, a CNS-expressed HDAC, which also plays a crucial role in cognitive function by influencing the repression of genes involved in synaptic plasticity and memory creation [72].

Electrically induced seizures in a different animal model of epilepsy revealed alterations in histone H3 and H4 acetylation at the CREB promoter in the rat hippocampus. CREB, a key transcriptional factor, influences GABAa receptor expression in the hippocampus and is significant in epilepsy development [73].

MicroRNAs are involved in protein translation and also play a role in immune responses that regulate epilepsy. During the early stages of brain development, miRNA controls gene translation, which is vital for the correct formation and maturation of dendrites and synapses. Neuronal translation of proteins like the NMDA receptor subunit (Mir-125b) and morphological proteins (p250GAP) is regulated by miRNAs [74]. Three recent studies in animal models indicate that epilepsy is linked to aberrant protein production, including substantial transcriptional inhibition. The studies observed that rats with induced status epilepticus showed higher miRNA-132 levels in their hippocampus; miRNA-132 is known to have anti-inflammatory properties, and inflammation is implicated in the development of epilepsy [75].

Additionally, the expression of TLR4 increased in mice experiencing experimentally induced epilepsy, and miRNA such as Let-7i plays a role in regulating TLR4 levels. It is believed that HMGB1, released from damaged neurons, acts as the ligand for TLR4 and promotes epileptic seizures [76].

Current knowledge suggests that, in vitro, Mir-146a inhibits proteins that promote inflammation, including IRAK1/2 and TRAF6, as well as IL-1B, in a negative feedback loop, although its in vivo target in epilepsy remains undetermined [77].

Regardless of age, unprovoked seizures are the leading cause of epilepsy. The most prevalent causes of secondary epilepsy are brain vascular malformations and head traumas [67].

Specifically, all types of epilepsy are a major public health concern because extended seizures or status epilepticus (SE) can cause cell death, which can lead to varying degrees of brain damage. Despite advancements in anti-epileptic drugs, challenges remain; specifically, side effects and the chance of seizures returning after suspended medication [78].

#### 1.3.2. MGBA Alterations and Epilepsy

Epilepsy is not only an important neurological disease alone, but also has close interactions with the occurrence and progression of numerous human disorders. In addition to epilepsy, patients may also have osteoporosis, fractures, and cognitive problems, and are at increased risk of death from suicide, vascular diseases, sudden unexpected death, and pneumonia [79,80]. Recent findings show that the GM is actively involved in regulating the GBA through direct mechanisms via the vagus nerve, and indirectly, by modulating the ENS [81]. Evidence increasingly points to systemic modulation within this complex GI ecosystem, potentially impacting both immunity and neurological processes, which supports the MGBA concept. Variations in the GM can cause systemic immune activation, leading to CNS inflammation, and conversely, neurological problems can initiate systemic inflammation, directly influencing gut microbiota. Such modifications frequently lead to problems with neuronal excitability and epileptogenesis, which results in seizure risk and confirms the clinical significance of the MGBA in epilepsy. As previously stated, the central role of BBB permeability suggests that changes in BBB permeability allow metabolite and neuropeptide production by the GM and GABA, which affects brain function by reaching the CNS, which influences seizure thresholds during epilepsy [82].

**Figure 2:**
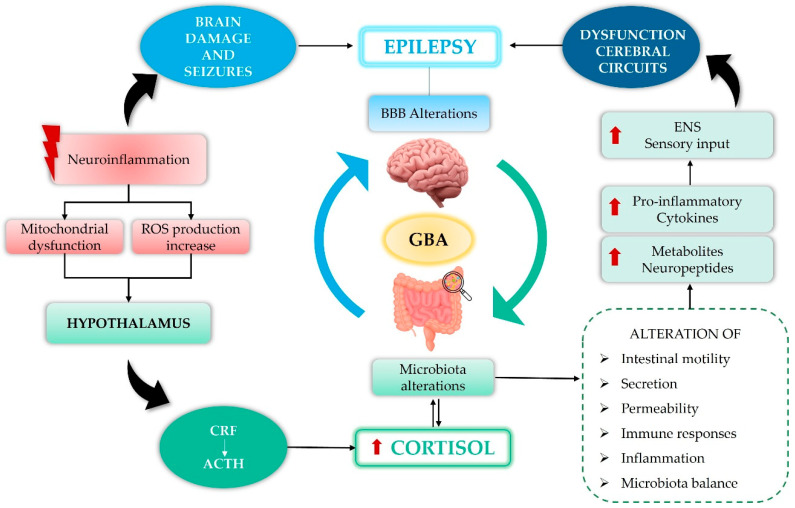
Communication between the GM and the central nervous system (CNS) occurs through the gut–brain axis, a bidirectional system that integrates neural, immune, and endocrine signals. Epilepsy is exacerbated by neuroinflammation, a consequence of mitochondrial dysfunction and increased reactive oxygen species (ROS), leading to brain damage and seizures. The hypothalamic–pituitary–adrenal (HPA) axis is activated by seizures, leading to corticotropin-releasing factor (CRF) release from the hypothalamus, followed by adrenocorticotropic hormone (ACTH) stimulation and cortisol production. By disrupting gut homeostasis, stress hormones modify the microbiota’s composition, permeability, motility, and immune responses. The increased production of microbial metabolites and neuropeptides, a result of this imbalance, allows them to access the central nervous system through a compromised blood–brain barrier (BBB). Pro-inflammatory cytokines and amplified signaling from the enteric nervous system further impair brain circuits, facilitating the onset of additional epileptic seizures. ↑: Increase; Abbreviations: Reactive Oxygen Species (ROS); Gut–Brain Axis (GBA); Blood–Brain Barrier (BBB); Corticotropin-Releasing Factor (CRF); Adrenocorticotropic Hormone (ACTH); Enteric Nervous System (ENS); Central Nervous System (CNS).

Thus, the MGBA is a multi-pathway network that is not completely understood. Thorough preclinical and clinical studies targeting these intricate pathways could help develop new therapeutic methods [82]. Indeed, 5-HT, vital for gut–brain interaction, acts as a key mediator; it is present in both the ENS and CNS [15]. Vagus nerve stimulation (VNS), used therapeutically since the 1980s for epilepsy, is particularly interesting. The activation of vagal afferent fibers changes the amounts of brain neurotransmitters like 5-HT, GABA, and glutamate, which could clarify its clinical success [83,84]. Changes to the gut barrier, for example, can affect the microbiota, causing more pro-inflammatory cytokines (e.g., TNF-α) and increased release of 5-HT by enteroendocrine cells (EECs) [85,86]. These processes contribute to sensitizing visceral afferent pathways, enhancing the impact of stress on motility, secretion, and permeability, which intensifies sensory input to the ENS [87]. Both in vivo and clinical research have demonstrated that prolonged stress can cause the up-regulation of central stress-response circuits, negatively affecting visceral and affective functions. Chronic stress is indeed related to the onset or aggravation of symptoms of irritable bowel syndrome (IBS) [88]. Patients suffering from IBS frequently show stress-induced alterations in gastrointestinal motility, intestinal sensitivity, autonomic regulation, and HPA axis activity [89]. Under physiological conditions, intestinal peptides maintain a synergy with the brain via homeostatic regulation; however, if the brain–gut axis malfunctions, this balance could be disrupted, resulting in irregular physical and pain neurotransmission. Constant increases in sensory signals may impact mood, anxiety, fear, and emotions [90,91], and epileptic seizures can be triggered by stress, which the HPA axis regulates [92]. Hormones affect epileptic activity differently; glucocorticoids rise in epileptics, and anticonvulsant deoxycorticosterone offers protection. Glutamate, along with CRF and corticosterone, may contribute to seizures by enhancing excitatory neurotransmitters (Figure 2) [93,94,95].

Neuroprotective and anticonvulsant effects have been observed in some beneficial bacteria, such as Akkermansia muciniphila and Parabacteroides, which influence the levels of GABA and glutamate in the hippocampus. On the other hand, dysbiosis might affect the GABAergic balance, increasing the chance of seizures [96,97]. Bifidobacterium spp. has been shown to boost immune responses and also release GABA, which can cross the BBB and affect the CNS. In vivo, Akkermansia and paramycetes colonization led to reduced glutamate and increased GABA in the hippocampus, with anticonvulsant effects [98]. Enterocromaffin cells in the gut produce roughly 90% of the body’s 5-HT, but the microbiota could highly influence its production [99,100]. The 5-HT is synthesized from tryptophan by some bacterial strains (e.g., Lactococcus, Lactobacillus, Escherichia coli, and Klebsiella) using tryptophan synthetase. In addition, studies show that sporigenic bacteria in the gut of mice and humans promote 5-HT synthesis in enteric cells via changes to the enzyme TPH1 [99]. In an in vivo study, reserpine-induced 5-HT depletion increased seizure susceptibility due to a lower epileptogenic threshold [101]. This implies that gut bacteria might influence gut and brain electrical function by controlling 5-HT, also affecting immune–inflammatory reactions. Both epilepsy patients and animal models display lower brain N-acetyl aspartate (NAA), which implies that the neuronal metabolism might be altered [102,103]. Indigestible fiber is processed by certain gut bacteria, including Firmicutes and Bacteroidetes, which then create SCFAs, which are responsible for microglia maturation and function. Increased seizure risk is connected to alterations in microglial function and BBB permeability [104,105]. Patients with DRE show significant differences in their microbiota when compared to those with drug-sensitive epilepsy or healthy individuals, which is interesting [106,107,108]. A study found that GM changes and SCFA reductions occurred before seizures in post-traumatic epilepsy, suggesting a connection between gut issues and seizure likelihood [109]. In genetic models, like the Wistar Albino Glaxo from Rijswijk (WAG/Rij) rats, which spontaneously develop seizures, studies showed lower brain SCFAs; however, butyrate helped decrease seizures, improve mitochondria, and increase seizure threshold [110]. Additionally, propionate supplementation demonstrated neuroprotective actions in the hippocampus, lessening mitochondrial damage and increasing seizure latency [111].

While the interconnection between gut microbiota/epilepsy and epigenetic modifications is still developing, it is hypothesized that microbial metabolites, including SCFAs, act as signaling molecules capable of influencing host epigenetic modifications [112]. These modifications can subsequently affect the genes that control nerve cell activity and inflammation. It is recognized that epigenetic modifications are crucial in the development of epilepsy. A potential strategy for preventing or treating epilepsy involves targeting these mechanisms, possibly through diet-induced, microbiome-mediated alterations [113].

Indeed, epigenetic changes, driven by the interplay of environmental risks and genetic vulnerabilities, are the source of significant phenotypic variation in neuropsychiatric disorders, without altering the DNA sequence itself [114]. According to evidence, an imbalanced gut microbiome and heightened gut permeability could be harmful, enabling the entry of molecules like pro-inflammatory cytokines and chemokines into the bloodstream, which may be neurotoxic and improperly activate the immune system [115].

The objective of this relationship is to enhance the immunity barrier and thereby control the microorganisms [116]. Clinical studies suggest a potential connection between neuropsychiatric disorders like epilepsy and alterations in gut microbiota composition and the gut–brain axis. Nevertheless, whether this link is directly correlated or proportional to the disorders’ severity is still under investigation [117]. The purpose of the ongoing observational study EPiGUT (ClinicalTrials.gov ID NCT07253701) is to understand the link between the gut and oral microbiota and various epilepsy types, and to determine if they affect how well medications for seizures function.

## 2. Endogenous and Exogenous Antioxidant Activity in Neurodegenerative Disease and Epilepsy

The growing understanding of free radicals’ role in epilepsy has prompted research into natural antioxidants for their potential to protect against seizure-related damage. In addition, the blood–brain barrier (BBB) is often damaged by excessive ROS production, resulting in a “leaky brain” condition. Indeed, BBB impairment leads to the infiltration of neurotoxic agents, white blood cells, and inflammatory cytokines into the brain, thus intensifying neuroinflammation and leading to epilepsy [118]. It has been demonstrated that these natural compounds positively impact epilepsy, specifically by decreasing convulsive behavior and alleviating brain oxidative stress. Particularly, vitamin D reduces mitochondrial ROS by increasing astrocytic glutathione and activating γ-glutamyl transpeptidase, whereas vitamin E targets lipid peroxidation chains and enhances endogenous antioxidants like SOD and catalase in preclinical settings [119].

### 2.1. GSH Levels in Epilepsy

The onset of neurodegenerative disease is currently unexplained, and cognitive function often declines after many nervous system disorders. In these neurodegenerative processes, a continuous loss of neurons is found, where ROS accumulation represents a critical step [120,121]. However, the death of neurons caused by trauma, ischemia, inflammatory lesions, excitotoxicity, and excessive ROS may be a trigger for the degenerative process in several diseases, including AD, Parkinson’s disease (PD), Huntington’s disease (HD), amyotrophic lateral sclerosis (ALS), Friedreich’s ataxia (FRDA), and epilepsy [122]. Epilepsy is strongly associated with oxidative stress, an imbalance between free radicals and the body’s defenses, which may cause seizures and brain damage, and is linked to a significantly impaired GSH system [58]. Recent research indicates that changes in GM are linked to epilepsy. The specific mechanism is still unknown; however, modifying the GM has a positive effect on the brain by regulating inflammation and oxidative stress [123,124].

The brain’s primary antioxidant, GSH, can be depleted by chronic seizures, creating a destructive feedback loop of oxidative harm and nerve cell problems. Indeed, reduced GSH levels often accompany diseases involving oxidative stress, increasing cellular damage [125]. The production of GSH in neurons is constrained by cysteine; using cysteine-metabolizing compounds as prodrugs could raise neuronal GSH levels. The involvement of GSH in neuronal diseases was first described in the neuronal ceroid lipofuscinoses (NCLs), which is known as Batten disease. Brain cells produced intracellular GSH from 0.2 to 10 mM levels. Reduced GSH levels may increase both oxidative stress and excitotoxicity, which could cause neuronal death. More evidence suggests that mitochondrial dysfunction is involved in causing and sustaining seizures. Animal models of temporal lobe epilepsy (TLE) and human patients have shown GSH depletion [126,127]. Further studies imply that more mitochondrial GSH might alter seizure chances and possibly make GSH an anticonvulsant drug. In fact, disruptions to GSH homeostasis and changes in GSH-dependent enzyme functions are often linked to the start and advancement of brain diseases like epilepsy. Reports suggest that this condition often relates to increased oxidants and alterations in the antioxidant system [128]. According to an in vivo study, pentylenetetrazol (PTZ) kindling raised adenosine deaminase (ADA) activity, but GSH treatment lowered it. This suggests that adenosine triphosphate (ATP) could be degraded. Compared to the control and sham groups, the PTZ kindling group showed high ADA activity. Increased purine catabolism could be linked to high ADA activity in mouse brain tissue during PTZ kindling seizures. Data imply that GSH treatment could prevent ATP degradation through a reduction in ADA, consequently influencing adenosine levels, which suggests that the antioxidant ability of GSH might not be the only reason behind the anticonvulsant effect of GSH [129]. Epilepsy, gut bacteria, and GSH are linked through the GBA. Lower GSH levels have been seen in epilepsy, and increasing them could prevent seizures. The KD increased levels of antioxidant and anti-inflammatory metabolites in the serum and hippocampus, including GSH, glycine, and N-acetyldopamine [130]. Previous research indicated that patients with epilepsy have lower cerebral GSH, and using the KD elevates brain GSH in patients with difficult-to-treat epilepsy [131]. The following studies have shown evidence of an interaction between ROS/reactive nitrogen species (RNS), GSH depletion, and neuroinflammation in diverse epilepsy models: 1) In rat electrical SE models, antioxidant treatments (N-acetil cysteine (NAC), sulforaphane) lowered oxidative stress. This reduction helped to lessen neuroinflammation, which could prevent seizure activity [132]; (2) Using a catalytic antioxidant to scavenge ROS lowered SE-induced pro-inflammatory cytokine production in a rat pilocarpine SE model [133]; (3) The KD has demonstrated strong anti-inflammatory effects in animal seizure models, which are partially attributed to enhanced tissue GSH levels [134]; (4) Acute seizures were observed in Theiler’s murine encephalomyelitis virus (TMEV)-infected mice, an infection-induced TLE model, and this correlated with impaired GSH redox status [135]. Overall, neuroinflammation involves oxidative stress and GSH depletion together [136]. Normally, ROS/RNS species are signaling molecules, but if there are too many, they can ruin vital thiol-based redox switches, which control neuronal excitability and neuroinflammation [137]. GSH deficiency can worsen the dysregulation of these pathways due to its antioxidant role. On the other hand, critical aspects of neuroinflammation, such as microglial activation, inflammatory pathway dysregulation, and cytokine/chemokine release, can cause oxidative stress and elevate seizure risk [138]. In addition to their role in oxidizing cellular macromolecules, ROS also function as signals, activating transcription factors like mitogen-activated protein kinase (MAPKs), nuclear factor kappa-light chain enhancer of activated B cells (NF-κB), and activator protein (AP-1), which are important in inflammation. These three pathways—MAPKs, NF-κB, and AP-1—are considered the “Holy Trinity” of intracellular signaling responsible for inflammation. These act as the primary control points, converting external threats (such as bacteria, stress, or injuries) into a cellular reaction that generates inflammatory substances [139].

Thus, comprehending the cellular and molecular actions in neuroinflammatory activation in epilepsy will help create new therapeutic approaches for managing epilepsy.

### 2.2. Coenzyme Q10 (CoQ10) Insufficiency and Epilepsy

CoQ10 is a strong antioxidant that protects cells from oxidative damage via the inhibition of some enzymes [140].

However, it is unclear how CoQ10 affects the seizure characteristics of epilepsy. New insights were revealed in a study regarding the connection between low CoQ10 and epileptic seizures. It demonstrated, for the first time, decreased CoQ10 plasma levels in patients, with a subsequent more frequent and longer-lasting epilepsy.

It seems that endogenous antioxidants and their repair capacity, which normally overcome the increased production of oxidants in cells, are reduced in ES patients.

Low CoQ10 levels are also linked to different neurological diseases, including stroke, neurodegeneration, and cerebellar ataxia, as well as a variety of other brain disorders [141,142]. Insufficient CoQ10 levels may cause increased electron transport to oxygen, resulting in a large production of O_2_^−^ in mitochondria [143]. Increased ROS and reduced ATP production could then injure the cell’s components. However, in the former, the impact of low CoQ10 is more significant in causing the diseases. On the other hand, higher levels of free radicals and fewer antioxidants have been associated with the onset of epilepsy [144]. Evidence from this study links CoQ10 levels to epilepsy’s frequency and duration. According to Yiş et al. (2009), CoQ10 deficiency elevates the risk of another seizure [145]. CoQ10 deficiency, whether complete or partial, dramatically boosts electron transfer to mitochondrial oxygen, causing excess O_2_^−^ free radicals. According to in vivo research, free radicals contribute to seizures, and antioxidants might alleviate oxidative stress indicators and reduce seizure activity [146].

Moreover, CoQ10, by scavenging antioxidants, prevents lipid peroxidation. In this context, animal seizure model studies reveal that treating epileptic rats with CoQ10 offers neuroprotection. This is achieved through eliminating free radicals, lowering lipid peroxidation, and decreasing nitrite, thereby mitigating seizure severity [147]. Pretreating with CoQ10 during the acute phase of pilocarpine-induced seizures has also been shown to reduce lipid peroxidation and increase antioxidant factors, lowering oxidative stress [147].

### 2.3. Nutritional and Nutraceutical Approach in the Management of Epilepsy by Microbiota Modulation

**Figure 3:**
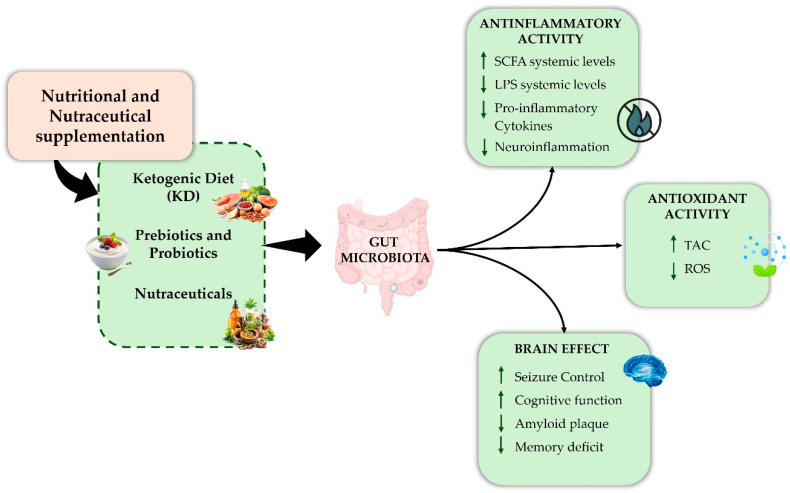
A nutritional and nutraceutical approach to neuroinflammation and epilepsy management through microbiota modulation. The ketogenic diet (KD), functional foods such as prebiotics and probiotics, and nutraceuticals represent potential complementary approaches in the management of neuroinflammation through the modulation of the gut microbiota. Nutritional and nutraceutical supplementation exerts anti-inflammatory activity by increasing systemic levels of short-chain fatty acids (SCFAs), reducing systemic levels of lipopolysaccharides (LPS) and pro-inflammatory cytokines, thereby leading to an attenuation of neuroinflammatory processes. They also have antioxidant activity, resulting in a reduction in reactive oxygen species (ROS) and a concomitant increase in total antioxidant capacity, leading to an overall decrease in oxidative stress. In the brain, modulation of the GM is also related to neuroprotective effects, contributing to the control of seizures, improving cognitive function and memory deficits, along with reducing amyloid plaque levels. ↑: Increase; ↓: decrease. Abbreviations: ketogenic diet (KD); short-chain fatty acids (SCFAs); lipopolysaccharides (LPS); reactive oxygen species (ROS); total antioxidant capacity (TAC).

The KD, prebiotics, probiotics, and nutraceuticals may help manage neuroinflammation through GM modulation [148,149]. Supplements with nutrients and nutraceuticals reduce inflammation by increasing SCFAs, lowering lipopolysaccharides (LPS) and cytokines, thus lessening neuroinflammation [150]. These compounds exhibit antioxidant activity, reducing ROS and enhancing antioxidant capacity, thus decreasing systemic oxidative stress. By influencing gut microbiota, the brain gains neuroprotective benefits through seizure control, cognition and memory amelioration, and amyloid plaque reduction (Figure 3).

#### 2.3.1. KD

Genetic factors, age, region, and diet can all affect GM composition [148]. The ketogenic diet (KD) has been widely used in the treatment of refractory epilepsy, with patients reporting positive results [149]. For example, 16S ribosomal ribonucleic acid (16S rRNA) sequencing studies have explored how the KD impacts the GM in mitochondrial epilepsy patients, suggesting it may enhance seizure control. This is thought to occur through changes in fatty acid metabolism, activation of the cyclic AMP (cAMP) signaling pathway via adenylate cyclase 3 (ADCY3), and increased neuronal inhibition. Akkermansia and Parabacteroides significantly increase in mouse models within four days of diet initiation. These microbes, when used for gnotobiotic colonization, protect against seizures in germ-free or antibiotic-treated mice [89,90,91,92,93,94,95,96,97,98,99,100,101,102,103,104,105,106,107,108,109,110,111,112,113,114,115,116,117,118,119,120,121,122,123,124,125,126,127,128,129,130,131,132,133,134,135,136,137,138,139,140,141,142,143,144,145,146,147,148,149,150,151]. Reduced γ-glutamyl transpeptidase (GGT) activity causes this effect, decreasing the production of γ-glutamyl amino acids like γ-glutamyl-leucine. Since these amino acids cross the blood–brain barrier through distinct transport mechanisms and participate in the synthesis of glutamate and GABA, their reduction increases the GABA/glutamate ratio in the brain and attenuates neuronal excitability [151,152,153,154]. Carbohydrate restriction may be linked to a decline in beneficial gut bacteria such as Bifidobacteria, Eubacterium rectale, and Dialister, while potentially increasing Actinobacteria and Escherichia coli, according to pediatric research. Functional analysis indicates depletion of the bacterial pathways involved in carbohydrate degradation [155,156]. A recent review highlighted a close relationship between the microbiota and GBA, clarifying the understanding that restoring the GM through ketoacidosis, probiotics, and FMT can improve DRE [157]. The ketogenic diet has demonstrated a beneficial impact on gut SCFA levels, especially with the inclusion of leafy greens, berries, and nuts [158]. After adhering to a KD, the study observed that 50% of the participating children had reduced seizures, and 10% achieved complete remission [159,160]. Traditional protocol rigidity and frequent adverse effects prompted the creation of alternative ketogenic formulations for better tolerability and adherence. Current primary dietary approaches consist of the classic ketogenic diet (cKD), KD, medium-chain triglyceride (MCT) diet, modified Atkins diet (MAD), and low glycemic index treatment (LGIT). Directly affecting neuronal excitability is supported by the experimental findings regarding ketone bodies. For example, acetone causes neuronal hyperpolarization and decreases excitability by activating K2P channels, which are essential for resting membrane potential. Neurotransmitters like glutamate, norepinephrine, and adenosine can be influenced by ketone bodies, which also enhance mitochondrial function; furthermore, they can exert epigenetic effects by regulating gene expression associated with seizure susceptibility, reducing DNA methylation through elevated levels of adenosine and histone hyperacetylation.

Medium-chain fatty acids have demonstrated a greater ability to influence phosphoinositide signaling in model organisms like Dictyostelium compared to valproate [161,162,163,164]. Decanoic acid might prevent seizures by blocking AMPA receptors in a noncompetitive manner, a proposed additional mechanism for its potential anticonvulsant effects. Decanoic acid reduces postsynaptic excitatory currents, acts as a non-competitive antagonist of amino-3-hydroxy-5-methyl-4-isoxazolepropionic acid (AMPA) receptors, and activates the nuclear receptor Peroxisome Proliferator-Activated Receptor gamma (PPARγ), thereby promoting mitochondrial proliferation and activation of the respiratory complex. Decanoic acid also enhances the anticonvulsant effect of valproate and, when combined with octanoic acid, can produce synergistic benefits [165,166]. Less frequently described mechanisms include the activation of voltage-dependent potassium channels by polyunsaturated fatty acids, increased production of brain-derived neurotrophic factor (BDNF) mediated by AMP-activated protein kinase (AMPK)/mTOR pathways, and remodeling of the gut microbiota, which increases the GABA/glutamate ratio in the hippocampus. The AMPK/mTOR pathway acts as a key metabolic regulator, with AMPK detecting low energy levels and subsequently inhibiting mTOR, which promotes growth. AMPK activation leads to the direct inhibition of mTORC1. This occurs via TSC2 phosphorylation or Raptor inhibition, subsequently activating autophagy and suppressing anabolic metabolism [167,168,169,170]. Clinical and observational studies confirm the efficacy of KD variants in adults with drug-resistant epilepsy. The modified Atkins diet (MAD) has demonstrated a moderate reduction in seizures (>25%) in patients with refractory focal epilepsy, as well as improvements in quality of life despite its demanding regimen [171,172]. The KD has also proven effective in cases of super-refractory status epilepticus, with seizure resolution rates of up to 82% and discontinuation within 3–25 days. With the classic KD, MCT, MAD, and LGIT, numerous studies have consistently documented decreased seizure frequency and intensity, as well as enhanced mood and quality of life [171,173]. Additionally, the MAD, MCT, and LGIT diets are more appealing and easier to follow than the classic KD, leading to fewer dietary limitations and less severe digestive issues. However, the links between these metabolic changes and treatment success are still unclear, and further studies are needed to clarify the correct mechanisms underlying these findings.

#### 2.3.2. Functional Food

Several studies have shown that the GM plays a crucial role in regulating various processes of the CNS via the MGBA. The GBA involves various ascending and descending pathways connecting the CNS, enteric nervous system, gut, and its microbiota. Additionally, the MGBA regulates gastrointestinal homeostasis and influences higher emotional and cognitive functions [174,175]. Based on current understanding, the brain creates and sends substances and signals affecting the GM, regulating the gastrointestinal tract, while neurotrophic substances from the gut may impact brain functions and behaviors [176]. According to new data, a connection between the GM and epilepsy has been demonstrated, suggesting a potential impact on neuron overactivity, seizures, and epileptogenesis. This promotes epileptogenesis and modifies inflammation’s pro-excitation via peripheral inflammation, which may manifest as neuroinflammation in the CNS [174,177]. Research on patients suffering from epilepsy (PWE) indicates a shared pathological link between epilepsy and its comorbidities. Intestinal dysbiosis may be a common factor, making it a possible treatment target [178]. Several preclinical studies found that treating animals with probiotics and prebiotics can enhance their cognitive function by strengthening gut barriers and the BBB, also by managing gut inflammation. These compounds rebalance the GM and promote the proliferation of SCFA-producing species, resulting in an increase in their systemic levels [179]. Reducing systemic LPS and enhancing biological barriers could decrease peripheral inflammation and glial activation, which may reduce the progression of neurodegeneration [180]. In addition, probiotics and prebiotics play a role in the restoration of neurotransmitter systems, with beneficial effects observed in several models of neurodegenerative diseases. They also play a crucial role in two main psychiatric effects, depression and anxiety [53,54,181,182,183,184,185,186].

##### SCFA Supplementation

**Figure 4:**
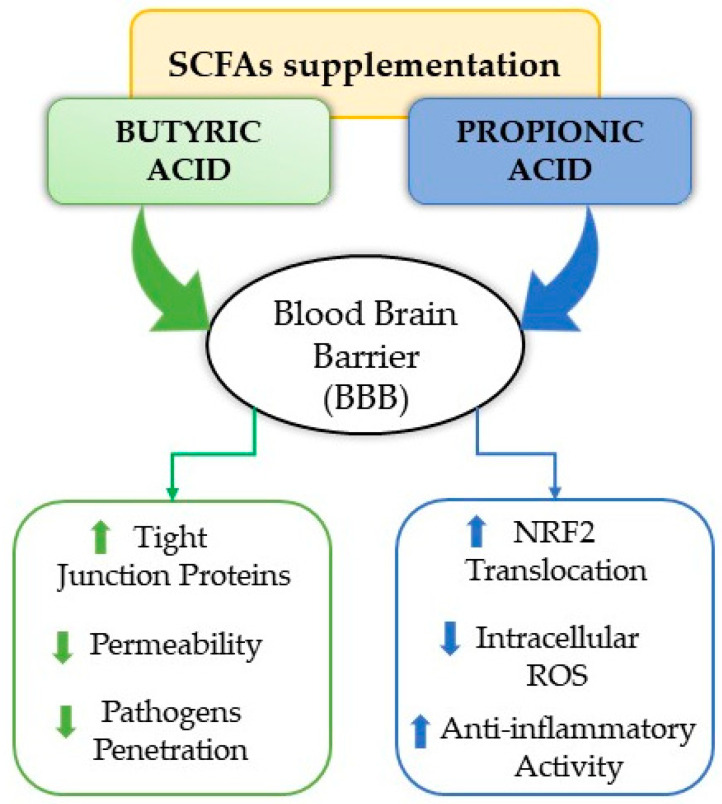
SCFA supplementation plays a key role in regulating blood–brain barrier (BBB) permeability and systemic inflammation. Butyric acid increases the expression of tight junction proteins in vascular endothelial cells, thereby reducing BBB permeability and limiting the penetration of pathogens and inflammatory mediators into the central nervous system (CNS). Propionic acid promotes nuclear translocation of the nuclear factor erythroid 2 (NRF2) transcription factor and reduces intracellular levels of reactive oxygen species (ROS), protecting the BBB from oxidative stress and inflammation. ↑: Increase; ↓: decrease. Abbreviations: Blood–Brain Barrier (BBB); Reactive Oxygen Species (ROS); Short-Chain Fatty Acid (SCFA); Nuclear Factor Erythroid 2 (NRF2).

The main components of SCFAs include acetic, propionic, and butyric acid [187]. Particularly, butyric acid and propionic acid play a key role in regulating BBB permeability and systemic inflammation. Butyrate increases the expression of tight junction proteins in vascular endothelial cells, thereby reducing BBB permeability and limiting the penetration of pathogens and inflammatory mediators into the CNS [188,189]. Propionate, on the other hand, promotes nuclear translocation of the nuclear factor erythroid 2 (NRF2) transcription factor and reduces intracellular levels of ROS, protecting the BBB from oxidative stress and inflammation (Figure 4) [190].

Supplementation with SCFAs can counteract these effects by improving tight junction proteins, such as claudin-1, zona occludes 1 (ZO-1), and occluding, which strengthen the intestinal mucosa and lower permeability [191,192]. In an in vivo model of PTZ-induced epilepsy, butyrate treatment had major anti-epileptic effects, which were linked to strengthening the intestinal barrier and lowering colic inflammation [193]. Findings suggest that probiotics might help to prevent or treat epilepsy; this potential is due to the ability of probiotics to modify the composition of the GM and to affect the nervous system [194]. Indeed, research has shown that active Bifidobacterium tripartitum bacteria could reduce the death rate of hippocampal neurons during epileptic seizures in mice. This is achieved by producing butyrate, which inhibits the activation of the cyclic GMP-AMP synthase-stimulator of interferon genes (cGAS/STING) pathway, and by decreasing the expression of Bcl2-associated X (Bax) and cleaved caspase-3 proteins [195]. In particular, the cGAS-STING pathway is a vital innate immune response that identifies DNA in the cytoplasm, signaling viral infections, bacterial incursions, or cell damage, thereby triggering inflammation and type I interferon release. The interaction of cGAS with double-stranded DNA (dsDNA) produces cGAMP, which activates STING. This activation then leads to IRF3 and NF-*κ*B being turned on, ultimately promoting immune surveillance, autophagy, and cellular senescence [196].

Furthermore, an in vivo study demonstrated the correlation between increased GABA levels and probiotics, particularly Lactobacillus and Bifidobacterium, due to their specific enzyme content. Studies show SCFAs have strong anti-epileptic effects in mice with colitis, but not in those without. Seizures were more likely with colitis; however, butyrate and alpha-lactoalbumin were the only anti-inflammatories with anti-epileptic effects [193]. An increase in total antioxidant capacity (TAC) and a reduction in total oxidative stress (TOS) were also observed in the hippocampus, suggesting that probiotics might exhibit neuroprotective effects against damage induced by free radicals acting in the pathogenesis of epilepsy [197].

##### PUFA Supplementation

**Figure 5:**
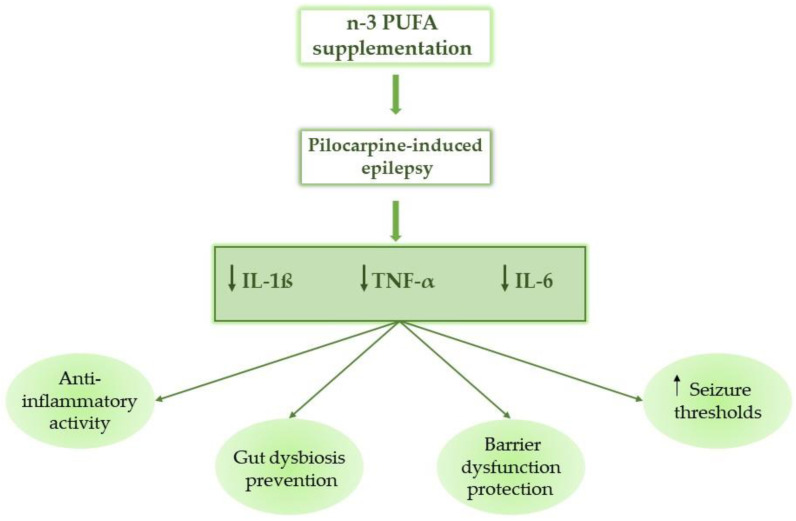
Potential anti-inflammatory effect of n-3 PUFAs in the management of epilepsy. In an experimental model of pilocarpine-induced epilepsy, Omega-3 polyunsaturated fatty acid (n-3 PUFA) supplementation reduced pro-inflammatory cytokines, such as interleukin-1 beta (IL-1ß), tumor necrosis factor alpha (TNF-α), and interleukin-6 (IL-6), in the blood. Indeed, these could raise seizure thresholds, exhibit anti-inflammatory activity, and potentially protect against intestinal dysbiosis and barrier dysfunction. ↑: Increase; ↓: decrease. Abbreviations: Omega-3 polyunsaturated fatty acids (n-3 PUFAs); interleukin-1 beta (IL-1ß); tumor necrosis factor alpha (TNF-α); interleukin-6 (IL-6).

A clinical investigation has revealed a possible therapeutic effect of fish oil in epileptic patients. This effect is related to omega-3 polyunsaturated fatty acids (n-3 PUFAs). In this experimental model of pilocarpine-induced epilepsy, rats treated with omega-3 fatty acids showed a reduction in pro-inflammatory cytokines in the blood, such as interleukin-1 beta (IL-1ß), tumor necrosis factor-alpha (TNF-α), and IL-6, thus confirming the potential anti-inflammatory effect of n-3 PUFAs in the management of epilepsy (Figure 5) [198].

Specifically, Epi rats showed a notable rise in linoleic acid metabolism. Therefore, the biosynthesis of unsaturated fatty acids is a metabolic difference between Epi and No-Epi rats. As an omega-6 PUFA, linoleic acid becomes arachidonic acid, which then produces prostanoids, while prostaglandins are specifically involved in neuroinflammation and oxidative stress, which are both common in epilepsy [199,200]. PUFAs include omega-3 PUFAs that are anti-inflammatory and could protect against gut dysbiosis and barrier dysfunction [201]. These findings show that lipid metabolism is altered in epileptic rats, which should be further explored through targeted lipidomics. Finally, Omega-3s can raise seizure thresholds and reduce inflammatory mediators often elevated in epilepsy patients, and linolenic acid is neuroprotective, preventing seizures and neuronal death [202].

##### Vitamins

**Figure 6:**
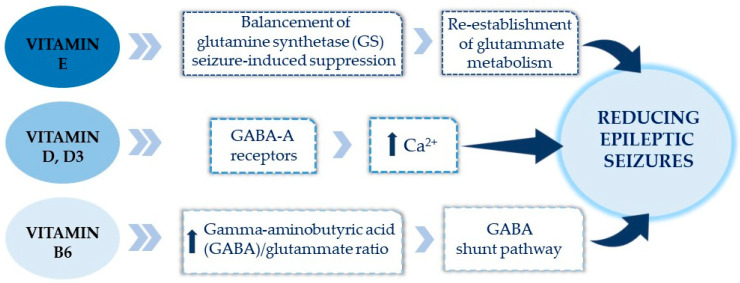
Vitamin supplementation (vitamins B6, D, D3, E) could be crucial in reducing epileptic seizures. Vitamin E, administered after status epilepticus (SE), was able to balance the seizure-induced suppression of glutamine synthetase (GS) and re-establish glutamate metabolism. Vitamin Ds, including vitamin D3, functioning as neuromodulators, involve GABA-A receptors by increasing calcium. Vitamin B6 supplementation could increase the Gamma-aminobutyric acid (GABA)/glutamate ratio, playing a pivotal role in the GABA shunt pathway. ↑: Increase; Abbreviations: Status Epilepticus (SE); Glutamine Synthetase (GS); Gamma-aminobutyric acid-A (GABA-A).

There is documented evidence of anti-epileptic drug interactions in epilepsy treatment [203,204]. Pharmacokinetic processes influence the interactions during absorption, metabolism, and excretion. Phenobarbital and phenytoin interact with vitamin D through metabolic enzymes, leading to significant drug interactions and vitamin D deficiency. Vitamin D’s function as a neuromodulator involves GABA-A receptors, impacting calcium and potentially reducing seizures [205]. Thus, providing vitamin D might represent a key to managing epilepsy in young patients. Furthermore, a clinical study showed the effect of vitamin D3 supplementation on seizures. A high dose of vitamin D3 significantly reduced the number of seizures in patients with poorly controlled epilepsy [206]. A recent pilot study comparing the number of seizures experienced during the 90 days prior to treatment onset to the number of seizures experienced in the 90 days after treatment onset of vitamin D3 therapy in epilepsy demonstrated that vitamin D3 could reduce epilepsy seizures, without any reported cases of toxicity [207]. An anti-seizure effect has been demonstrated, showing that vitamin E, administered after SE, was able to re-establish glutamate metabolism by balancing the seizure-induced suppression of glutamine synthetase (GS), an enzyme that can decrease synaptic glutamate levels. Additionally, clinical studies support that B6 supplementation could improve epileptic seizures in patients suffering from this condition (Figure 6) [208,209].

#### 2.3.3. How Nutraceuticals Might Ameliorate Neuroinflammation and Seizures Through MGBA

Polyphenols, which are phytonutrients from plants, have garnered considerable interest because of their potential to treat neurological conditions and neuroinflammation. These compounds show multiple neuroprotective features, such as antioxidant, anti-inflammatory, and anti-amyloid qualities, that help to reduce the progression of neurodegenerative diseases, and they have been intensely investigated for their capacity to control inflammation by changing pro-inflammatory gene activity and impacting signal pathways, thus decreasing neuroinflammation and neuron death (Table 1). Polyphenols have also shown potential to affect cellular signals linked to neuron health, synaptic changes, and thought processes. Polyphenols primarily offer neuroprotection by influencing oxidative stress, a key factor in neuroinflammation [210]. Due to their strong antioxidant activity, polyphenols can neutralize ROS and reduce oxidative damage to neurons [211,212].

Researchers have analyzed the role of polyphenols, in particular flavonoids, positive allosteric modulators of GABA receptors, and found that polyphenols’ affinities for the benzodiazepine site are beneficial in enhancing neuronal inhibition and reducing the excitability associated with epileptic seizures [223]. These compounds appear to increase GABAergic transmission or reduce glutamatergic activity, thus contributing to a beneficial neurochemical balance. These findings confirm that research on flavonoids, as a potential treatment for epilepsy, particularly drug-resistant cases, is a growing trend of exploring natural compounds [223]. A recent study showed that Lippia origanoides essential oil (LOEO) treatment is effective in treating pentylenetetrazol-induced epileptic seizures in rats. Furthermore, LOEO exhibited a synergistic anticonvulsant effect combined with diazepam (DZP), enhancing efficacy while minimizing side effects [213]. More research highlighted the neuroprotective properties and cognitive-enhancing activities of benzyl isothiocyanate (BITC), a natural compound found in cruciferous vegetables. These effects have been observed in mice with chronic temporal lobe epilepsy induced by lithium-pilocarpine, specifically concerning learning, memory, and spatial cognition. Treatment with BITC increased the antioxidant capacity of hippocampal tissue by activating the nuclear factor E2-related factor/haem oxygenase 1 (NRF2/HO-1) signaling pathway. The Nrf2/HO-1 signaling pathway plays a crucial role in defending cells from oxidative stress, inflammation, and metabolic dysfunction. Nrf2 is a transcription factor that, upon activation, increases the production of HO-1, an enzyme involved in heme degradation and providing antioxidant, anti-inflammatory, and cytoprotective functions. Additionally, an increase in glutathione peroxidase (GSH-Px) activity and a reduction in malondialdehyde (MDA) content were observed [214]. A study found that tetrahydrocurcumin, a metabolite of curcumin, plays a neuroprotective role in neurodegenerative disorders by reducing oxidative stress, modulating neuroinflammation, activating autophagy, and inhibiting the mitochondrial apoptotic pathway [224]. In fact, curcumin can prevent Parkinson’s disease from progressing. Consequently, curcumin may also have anti-epileptic and neuroprotective effects by controlling the GBA’s equilibrium [220]. Resveratrol’s regulatory role in the GBA is backed by increasing evidence, and its mechanisms involve glucagon-like peptide-1 (GLP-1), the 5-HT system, and gut microbiome diversity. GLP-1 protects against neurodegenerative diseases like AD, PD, and stroke [225,226]. Resveratrol could enhance GLP-1 effects in the CNS and intestine by boosting silent information regulator 1 (SIRT1) and forkhead box, sub-group O (FOXO) gene activity. To enhance resveratrol’s efficacy in preventing amyloid-beta (Aβ) accumulation in the hippocampus and to address GM imbalance by controlling Alistipes, Helicobacter, Rikenella, Desulfovibrio, and Faecalibaculum, a small resveratrol–selenium–peptide nanocomposite was created using an ADs mouse model [215]. Epigallocatechin-3-gallate, a catechin (EGCG), showed a protective effect by changing the GM of Drosophila melanogaster with Phosphatase and Tensin Homolog (PTEN)-induced kinase 1 (PINK1) mutations in a prototype PD model [216]. Quercetin-3-O-glucuronide (Q3G), a flavonol, may counteract cognitive impairment caused by Aβ by reversing brain insulin resistance [221]. Silibinin and silymarin might improve memory issues and decrease amyloid plaques in precursor protein- presenilin- 1 (APP/PS1) mice. These polyphenols changed microbiota diversity and impacted the levels of specific AD-linked bacteria, suggesting silibinin/silymarin may fight AD via GM control [217]. Luteolin, found in celery, parsley, and thyme, inhibits microglial activation and lowers pro-inflammatory cytokines [218]. According to Charrière et al., apigenin from chamomile and parsley changes GABAergic activity, decreases amyloid plaques, and has anti-inflammatory properties [227]. Furthermore, anthocyanins, a type of water-soluble flavonoid that acts as a natural color pigment in colorful plants like berry fruits and vegetables, have garnered considerable interest for their neuroprotective effects on the CNS. In particular, berries such as blueberries and raspberries contain anthocyanins, which alleviate oxidative stress, lessen neuroinflammation, and improve synaptic plasticity, thus reducing both neural cell apoptosis and neuronal inflammation and improving microglia vitality [228].

Current reports indicate anthocyanins may impact the gut microbiota, subsequently influencing the CNS. Indeed, gut neurotransmitter synthesis pathways might impact brain neuronal activity and cognitive function [229].

Further research indicates that anthocyanins impact host tryptophan metabolism, generating metabolites that could modulate CNS inflammation. Consequently, anthocyanins can function as microbe–gut–brain axis mediators, thereby controlling neuroinflammation through gut microbial modification. Studies have also shown that anthocyanins can influence the gut microbiota, which in turn affects the production of metabolic molecules (e.g., tryptophan, SCFAs) and harmful substances (e.g., LPS), enhancing BBB integrity and reducing neuroinflammation in nerve cells to treat neurodegenerative diseases [228].

Another study revealed that BB supplements enhanced spatial memory in underperforming animals while maintaining it in high performers. Latency was where these effects were clearest: BB-fed poor learners found the platform faster, BB-fed good learners did not slow down, and control-fed rats took longer. Average performers fed BBs had increased latency from pre- to post-test. The fact that raspberry and BB improved performance in those with poor results is expected, as this group had the most room to improve [222]. In addition, pterostilbene in grapes and blueberries has been observed to boost antioxidants, affect sirtuins, and lower brain inflammation [230].

The hesperidin in citrus fruits (oranges, lemons) elevates antioxidant defenses, fights neuroinflammation, and improves cognitive function [231]. Strawberries, apples, and onions contain Fisetin, a compound that acts as an antioxidant, reduces inflammation, and affects aging processes [232]. Oxidative stress is lessened, estrogen receptors are adjusted, and neuroinflammation is stopped by genistein, which comes from soybeans and legumes [233]. Citrus naringenin reduces inflammation, oxidative stress, and prevents Aβ aggregation [219]. Epileptic activity is accompanied by multiple neurological comorbidities, whereas secondary seizures are commonly observed in multiple neuropathological contexts. The anti-inflammatory and antioxidant properties of polyphenols make them potentially attractive in the treatment of both epilepsy and epilepsy-associated disorders [234]. These bioactive compounds might regulate several molecular cascades tied to diverse neurological disorders. As a result, investigating the potential of polyphenols to prevent symptomatic seizures is important [235]. Changes in epileptic patients’ microbiota could encourage seizures. More research is needed to understand how polyphenols directly affect the gut and benefit epilepsy. However, various anti-epileptic polyphenols affect the GBA, altering the human microbiota related to epilepsy [236]. Furthermore, the gut–brain axis highly influences the effect of polyphenols on neurotrophic factors [237]. Polyphenols are changed by the GM into active compounds, which then cross the blood–brain barrier and impact the CNS [238]. These interactions might improve cognitive function and mood by boosting BDNF and reducing neuroinflammation. Despite this enhanced range of traditional pharmaceutical treatments, the prevalence of refractory epilepsy in patients diagnosed with epilepsy remains at a high level, accounting for around 30% of cases. Gut microbiome dysbiosis is seen in epilepsy patients and animal models, implying that treatments that restore GM balance may be anti-epileptic candidates thanks to their antioxidant and anti-inflammatory activity. Therefore, a new method for treating epilepsy might involve using nutraceuticals to affect, primarily, gut microbiome dysfunction. Animal models are the preferred method for epilepsy studies, and even with advancements in studying epileptogenesis in animals, it is essential to remember the differences in brain damage between humans and animal models. Consequently, it is important to consider the translational limitations of animal model findings, which may not accurately reflect human conditions due to differences in anatomy, physiology, and pharmacology. Overall, supplementation with nutraceuticals could help bridge the gap until innovative therapeutic methods are developed [239].

### 2.4. FMT in Neurodegenerative Disease and Epilepsy

The gut–brain axis is a new research frontier in neurological disorders, with growing evidence that the intestinal microbiota can affect the CNS [240]. Furthermore, novel techniques for examining and altering the gut microbiome, such as metabolomics and FMT, have emphasized the significant influence of the human gut microbiota on neuroinflammation, as well as metabolic and neuroendocrine signaling pathways [241].

FMT involves introducing a healthy donor’s fecal solution into a recipient’s digestive system to repopulate the gut microbiome. Research indicates that FMT has been applied extensively to neurological disorders such as Alzheimer’s, Parkinson’s, autism, MS, and epilepsy, yielding beneficial outcomes [242].

However, only a small number of studies have examined FMT’s effect on epilepsy patients and animal models. Specifically, a clinical study showed a reduction in seizure frequency after three FMT sessions in a 17-year-old girl who had both Crohn’s disease and epilepsy. After twenty months of FMT, the patient’s seizures completely resolved, and they no longer required anti-epileptic drugs. Additionally, the Chron’s disease activity index, a measure of illness severity, decreased significantly from 361 to 131 points [243].

According to Citraro et al., FMT from non-transgenic rats can lower seizure frequency and duration in the WAG/Rij rat model of genetic absence epilepsy. The study showed that WAG/Rij rats receiving fecal transplants from non-transgenic animals treated with ethosuximide had a greater reduction in absence seizure frequency and duration compared to those receiving transplants from untreated animals [244].

In a 6-Hz corneal stimulation seizure model, an in vivo study investigated KD mechanisms. It was discovered that FMT from KD-fed donors to germ-free mice elevated seizure threshold, comparable to the KD’s own impact. However, transplanted fecal microbiota from control chow-fed donors into GF animals failed to offer the seizure protection seen with the KD [98].

Similarly, Medel-Matus et al. transferred fecal microbiota from stressed or sham-stressed donors to recipients whose commensal microbiota had been depleted. Using the rapid amygdala kindling model for epileptogenesis in rats, the effects of FMT on seizures were assessed. The study reported that sham-stressed recipients required fewer stimulations to achieve full kindling and experienced longer seizure durations compared to sham-stressed recipients given FMT from sham-stressed donors [245]. By contrast, fecal microbiota transplants from sham-stressed donors into stressed recipients resulted in an increased number of stimulations for full kindling and a shorter seizure duration, compared to stressed recipients receiving FMT from stressed donors. These findings suggest that stress and other conditions that sensitize the brain to seizures are affected by the gut microbiota [245].

## 3. Conclusions and Future Perspectives

In conclusion, the GM strongly impacts both brain development and associated disorders. Brain–gut axis interactions greatly involve gut microbes and their metabolites. Despite the early stage of gut–brain axis exploration, some fundamental circuits are starting to become apparent, and gut microbial signals might be necessary for specific neurodevelopmental pathways to respond. The literature extensively supports the ketogenic diet’s role in treating drug-resistant epilepsy. However, current research is limited by non-standardized studies, a focus on short-term results, possible patient compliance issues, and an incomplete understanding of the diet’s precise workings. Different mechanisms of action are suggested, such as metabolic shifts influencing neuronal excitability and neurotransmitter concentrations, enhanced mitochondrial efficiency, reduced neuronal damage, correction of sleep architecture, and alteration of the gut microbiome. According to the “ketomicrobiota” concept, the gut–brain axis is altered by the microbiome, leading to enhanced DRE outcomes. Although this concept holds promise, it remains quite novel, necessitating further investigation to fully elucidate the gut microbiome’s role in drug-resistant epilepsy [246]. The GM is key to the bioavailability and bioaccessibility of polyphenols, which are needed for absorption in the small intestine. Intestinal homeostasis depends on polyphenols’ metabolites, which regulate the intestinal barrier, improve immunity, influence signaling pathways, promote probiotics, and inhibit pathogen development. Previous research showed that the human GM converts naringin to naringenin, supporting the concept that fibers enhance the availability of bioactive compounds [247]. Indeed, findings show that micronizing polyphenols and, with greater stability, encapsulating them in fibers might improve naringenin and metabolites’ bioavailability, which are key modulators of systemic inflammation, hyperlipidemia, and oxidative stress [248,249]. Despite positive in vivo findings for nutraceuticals, their clinical use is limited by a lack of robust human studies and extensive trials. However, the evidence directly linking antioxidants from food to seizure risk is contradictory, and certain supplements, despite being perceived as safe, could potentially exacerbate seizures or harm the CNS. Higher doses, necessary for therapeutic effects and beyond normal dietary intake, might have detrimental interactions with conventional anti-seizure drugs (ASDs) [250]. Coated biocompatible probiotics demonstrate better gut environment resistance, as they can adhere to mucus, which enhances retention and facilitates intestinal colonization. Furthermore, they improve the controlled release of probiotic cells in the colon and might be useful for preventing and treating different chronic diseases at the cellular level. In particular, it has been highlighted that the development of well-designed, edible delivery systems could increase probiotic effectiveness in both preventing and treating colorectal cancer [251]. However, since studies on probiotics involve small, diverse samples (different strains, dosages) and lack long-term, reproducible results across all patients, there are no official guideline recommendations for their routine use in clinical practice. In light of the aforementioned preclinical and clinical investigations, and the altered gut microbial ecosystem in individuals with epilepsy, FMT might serve as a viable clinical treatment for epilepsy through SCFAs. Restoring decreased seizure thresholds, EIB-relevant gene expression, spontaneous inhibitory postsynaptic current frequency, and BBB integrity, while diminishing stress-induced proepileptic effects, was achieved by transplanting GM from healthy controls to seizure-induced animal models [252]. Clinical studies have only one report showing FMT’s anti-epileptic effects [252]. FMT, in the form of oral capsules or drinks, has nevertheless been successful in clinical trials for common epileptic comorbidities, including depression and autism spectrum disorder [253]. Clinical trials have not yet indicated any significant adverse effects from FMT, which is a very direct method for altering the GM [253]. More research is needed to address certain questions, even though healthy FMT has been shown to help with epilepsy, such as the ways FMT works to prevent epilepsy, the lasting power of its good effects, and the specific bacteria genera or phyla responsible. Animal models demonstrate that FMT’s anxiolytic effects are mediated by the vagus nerve, with vagotomy nullifying its impact in rats. However, a thorough investigation into the vagus nerve’s contribution to FTM’s impact on epilepsy is necessary. Additional studies are also necessary to investigate the role and the interplay between oxidative stress and the MGBA, which might be affected directly or indirectly by several intervention types (e.g., diet, supplements, drugs). Investigating these interventions and identifying biomarkers might give greater insight into epilepsy and provide new therapeutic approaches [254]. Additionally, despite pharmacological treatments being the main way to handle epilepsy, the KD, functional foods, and nutraceuticals are gaining attention as potential additional treatments [255]. In particular, the KD represents a valuable therapeutic strategy for pharmacoresistant epilepsy and refractory SE in adults. The next steps in researching ketogenic diets involve improving diet variations, examining combination therapies, conducting long-term safety research, measuring quality of life improvements, expanding international implementation, and developing consistent clinical guidelines for prescription and care. Its complex benefit/risk profile requires individualized assessment, careful clinical oversight, and consideration of the various dietary formulations to optimize adherence and therapeutic outcomes.

These compounds affect neuronal excitability, neurotransmitter release, and neuroinflammation, thus providing anticonvulsant effects. In addition, it might enhance the effectiveness of conventional anti-epileptic drugs while counteracting their adverse effects [234]. The challenge with clinical functional foods and nutraceuticals use is determining the right dosage and treatment approach. The correct dosage is key to preventing adverse effects and ensuring the best anti-epileptic results. Whether polyphenol uptake strategies need customization based on patient condition is unclear [255]. Based on the scientific evidence, the occurrence of epilepsy is related not only to the nervous system but also to the immune and metabolic systems. Various factors are involved in neurodegenerative protein accumulation, neurotransmitter imbalance, glial cell proliferation, nerve excitability, synaptic changes, neuronal voltage, ion channel mutations or variants of ligands, inflammatory reactions, oxidative stress, mitochondrial damage, and dysfunction of glycogen metabolism. Future clinical studies are needed to better understand how the GM affects epilepsy. Certain nutraceuticals might have probiotic effects, and the gut bacteria they impact could vary based on different illnesses; therefore, a new combined treatment idea using nutraceuticals and/or functional foods to supplement traditional therapies has been suggested. A synergic effect of nutraceuticals and functional foods could be interesting, perhaps offering a stronger therapeutic effect compared to using either alone. Further investigation is required into the synergistic effects of nutraceuticals, gut microbiota, and functional foods on epilepsy treatment, particularly regarding enhanced absorption [234,256]. Additionally, combining pro/prebiotics with FMT might lead to targeted GM therapies in the future, potentially customized to individual patient conditions [253]. Nonetheless, due to the substantial disparity between laboratory testing and clinical application, additional investigations are required to establish the long-term success and trustworthiness of FMT in individuals with epilepsy.

## Figures and Tables

**Table 1:** This table summarizes the main evidence highlighting how nutraceuticals might ameliorate neuroinflammation and seizures through MGBA.

Authors, Year	Aim of Studies	Types of Studies	Summary of Results	Refs.
Bastos de Araújo D, et al. 2023	To assess the anticonvulsant effect of Lippia origanoides essential oil (LOEO), diazepam (DZP), and their combination in suppressing and controlling pentylenetetrazol (PTZ)-induced seizures.	In vivo study	LOEO increased the latency time for the appearance of isolated clonic seizures without loss of the postural reflex. The animals had a more intense decrease in respiratory rate when combined with LOEO + DZPElectroencephalogram (EEG) recordings showed a reduction in firing amplitude in the LOEO-treated groups. Combining treatment with DZP resulted in increased anticonvulsant effects. Therefore, treatment with LOEO was effective in controlling seizures.	[213]
Xiaoyu C, et al. 2024	To investigate the neuroprotective effect of benzyl isothiocyanate (BITC) on a lithium-pilocarpine-induced temporal lobe epileptic mouse model.	In vivo study	BITC enhances cognitive function and motor ability in mice. BITC treatment plays a positive role in neuroprotection, especially in the cortex. The BITC treatment group, when compared to the EP group, showed enhanced transcription levels of nuclear factor erythroid 2 (NRF2), HO-1, and NQO1, along with increased glutathione peroxidase (GSH-Px) activity, and a decrease in malondialdehyde (MDA) content.	[214]
Li C, et al. 2021	A small resveratrol–selenium–peptide nanocomposite was designed to prevent beta-amyloid (Aβ) aggregate-induced neurotoxicity and to regulate the balance of GM disorder in aluminum chloride (ALCL3)- and d-galactose (d-gal)-induced AD model mice.	In vivo and in vitro study	Oral administration of TGN-Res SeNPs improves the following: 1. Cognitive disorder through interacting with Aβ and decreasing Aβ aggregation, effectively inhibiting Aβ deposition in the hippocampus; 2. Decreasing Aβ-induced reactive oxygen species (ROS) and increasing activity of antioxidation enzymes in PC12 cells. In vivo down-regulating Aβ-induced neuroinflammation via the nuclear factor kappa-light chain enhancer of activated B cells (NF-κB/mitogen-activated protein kinase/Akt signal pathway) in BV-2 cells. In vivo alleviating GM disorder, particularly with respect to oxidative stress and inflammatory-related bacteria.	[215]
Xu Y, et al. 2020	To assess behavioral outcomes, epigallocatechin-3-gallate (EGCG) was administered to the Drosophila melanogaster exhibiting PINK1 (Phosphatase and Tensin Homolog (PTEN)-induced putative kinase 1) mutations in a prototype Parkinson’s disease (PD) model.	In vivo study	PINK1 B9 mutant flies exhibited dopaminergic, survival, and behavioral deficits, which were improved by EGCG. EGCG treatment also altered the composition of the gut microbiota; when the microbiota was altered with antibiotics, the benefits of EGCG disappeared. Transcriptomic analyses identified the TotM gene as a key player in the response to EGCG and microbiota changes, and its deletion blocked the neuroprotective effect.	[216]
Shen L, et al. 2019	To explore the impact of silibinin and silymarin on behavioral and histological outcomes, including their modulation of the GM of precursor protein- presenilin- 1 (APP/PS1) transgenic mice.	In vivo study	Silibinin and silymarin administration could alleviate memory deficits and reduce the amyloid plaque burden in the brain of APP/PS1 mice in comparison with controls. Silibinin and silymarin administration tended to decrease the microbiota diversity and exhibited regulative effects in abundances on several key bacterial species associated with AD development.	[217]
Mugundhan V, et al. 2024	To investigate the impact of ferulic acid (FA) on acetylcholinesterase (AChE) enzyme activity and Aβ plaque growth in an in vitro model of Alzheimer’s disease (AD).	In vitro study	FA has the potential to be an AChE inhibitor, and also to reduce the incidence of amyloid beta plaque formation. FA exhibited a significant antioxidant property by the xanthine oxidase enzyme inhibitory effect.	[218]
Choi GY, et al. 2023	To explore the neuroprotective efficacy of naringin on long-term potentiation (LTP) in organotypic hippocampal slice cultures.	Ex vivo study	In hippocampal tissue slices, naringin dose-dependently increased field excitatory postsynaptic potential (fEPSP) and attenuated Aβ-induced fEPSP blockade in the CA1 area of the hippocampus. In Aβ-injected rats, naringin improved object recognition memory, avoidance memory, and spatial recognition memory. In the hippocampus, naringin attenuated Aβ-induced activation of cyclooxygenase-2 and Bcl2-associated X (Bax) and inhibition of Bcl-2, cAMP-Response Element Binding protein (CREB), brain-derived neurotrophic factor (BDNF), and TrkB.	[219]
Cui C, et al. 2022	To assess the potential of curcumin (CUR) to protect the nervous system in a mouse model of PD induced by 1-methyl-4-phenyl-1,2,3,6-tetrahydropyridine (MPTP).	In vivo study	CUR intervention effectively improved motor deficits, glial cell activation, and α-synuclein (α-syn) aggregation in MPTP-treated mice. CUR treatment led to a rapid increase in brain tyrosine and levodopa (DOPA) levels; these changes correlated with the abundance of Lactobacillaceae and Aerococcaceae. CUR exerts a protective effect on PD progression by modulating the gut microbiota-metabolite axis.	[220]
Xu M, et al. 2021	To examine whether Quercetin-3-O-glucuronide (Q3G) could enhance cognitive function in mice by regulating inflammation and insulin resistance (IR) in their brains.	In vivo study	Q3G attenuates neuroinflammation and brain IR in Aβ1-42-injected mice and alleviates apoptosis in Aβ1-42-treated SH-SY5Y cells by disrupting downstream insulin signaling. Q3G enhances Aβ accumulation and Tau phosphorylation, restores CREB and BDNF levels in the hippocampus, and reverses Aβ1-42-induced cognitive impairment. Furthermore, Q3G restores Aβ1-42-induced short-chain fatty acid (SCFA) reduction and dysbiosis.	[221]
Shukitt-Hale B, et al. 2019	To examine how consuming blueberries affects cognitive function in older rats, considering their existing cognitive function and inflammation.	In vitro and in vivo study	A significant reduction in latency in the radial arm water maze (RAWM) in poor-performing rats supplied with blueberries (BB) (*p* < 0.05), and it was maintained in good-performing rats supplied with BB. The high-performing control rats showed increased working and reference memory errors in the post-test compared to the pre-test (*p* < 0.05). Supplementation with blueberries did not change the good-performing rats. LPS-induced nitrite production and tumor necrosis factor-alpha (TNF-α) levels were reduced by BB supplementation.	[222]

LOEO: Lippia origanoides essential oil; DZP: diazepam; PTZ: pentylenetetrazol; BITC: benzyl isothiocyanate; GSH-Px: glutathione peroxidase; MDA: malondialdehyde; NF-κB: nuclear factor kappa-light chain enhancer of activated B cells; NRF2: nuclear factor erythroid 2; SIRT1: silent information regulator 1; ALCL3: aluminum chloride; AD: Alzheimer’s disease; EGCG: Epigallocatechin-3-gallate; PINK1: Phosphatase and Tensin Homolog (PTEN)-induced kinase 1; Q3G: Quercetin-3-O-glucuronide; APP/PS1: precursor protein-presenilin-1; FA: ferulic acid, AChE: acetylcholinesterase; Aβ: amyloid-beta; LTP: long-term potentiation; BB: blueberries; ROS: reactive oxygen species; TNF-α: tumor necrosis factor-alpha; CREB: cAMP-Response Element Binding protein.

## Data Availability

No new data were created or analyzed in this study.

## Abbreviations

16S rRNA	16s ribosomal ribonucleic acid
5-HT	Serotonin
AChE	Acetylcholinesterase
ACTH	Adrenocorticotropic hormone
ADA	Adenosine deaminase
ADCY3	Adenylate cyclase 3
ADNFLE	Autosomal Dominant Nocturnal Frontal Lobe Epilepsy
AD	Alzheimer’s disease
ALCL3	Aluminum chloride
ALS	Amyotrophic lateral sclerosis
AMPA	Amino-3-hydroxy-5-methyl-4-isoxazolepropionic acid
AMPK	AMP-activated protein kinase
ANS	Autonomic nervous system
AP-1	Activator protein
APP/PS1	Precursor protein–presenilin-1
ASMs	Anti-seizure medications
ATP	Adenosine triphosphate
Aβ	Amyloid-beta
Bax	Bcl2-associated X
BB	Blueberries
BBB	Blood–brain barrier
BDNF	Brain-derived neurotrophic factor
BITC	Benzyl isothiocyanate
CAE	Childhood absence epilepsy
cAMP	Cyclic AMP
cGAS/STING	Synthase-stimulator of interferon genes
CI	Chain complexes I
CIII	Chain complexes III
CNS	Central nervous system
CoQ10	Coenzyme Q10
CREB	cAMP-Response Element Binding protein
CRF	Corticotropin-releasing factor
d-gal	d-galactose
DNA	Deoxyribonucleic acid
DRE	Drug-resistant epilepsy
DZP	Diazepam
EE	Epileptic encephalopathy
EECs	Enteroendocrine cells
EEG	Electroencephalogram
EEs	Epileptic encephalopathies
EGCG	Epigallocatechin-3-gallate
ENS	Enteric nervous system
FA	Ferulic acid
FAT/CD36	Fatty Acid Translocase Cluster of Differentiation 36
FMT	Fecal microbiota transplantation
FOXO	Forkhead box, sub-group O
FRDA	Friedreich’s ataxia
FXR	Farnesoid X receptor
GABA	Gamma-Aminobutyric Acid
GABRA1	Gamma-Aminobutyric Acid Type A Receptor Alpha 1 Subunit
GABRG2	Gamma-Aminobutyric Acid Type A Receptor Gamma 2 Subunit
GBA	Gut–brain axis
GGE	Genetic generalized epilepsy
GGT	Gamma-Glutamyl Transpeptidase
GLP-1	Glucagon-like peptide-1
GM	Gut microbiota
GS	Glutamine synthetase
GSH	Glutathione
GSH-Px	Glutathione peroxidase
HD	Huntington’s disease
HPA	Hypothalamic–pituitary–adrenal axis
IBS	Irritable bowel syndrome
IDO1	Indoleamine 2,3-dioxygenase 1
IL-1ß	Interleukin-1 beta
IL-6	Interleukin-6
ILAE	International League Against Epilepsy
JME	Juvenile myoclonic epilepsy
K2P	Two-pore domain potassium channels
KD	Ketogenic diet
LAT1	Large Neutral Amino Acid Transporter 1
LGIT	Low Glycemic Index Treatment
LOEO	Lippia origanoides essential oil
LPS	Lipopolysaccharide
LTP	Long-term potentiation
MAD	Modified Atkins Diet
MAPKs	Mitogen-activated protein kinase
MCT	Medium-chain triglyceride diet
MCT1	Monocarboxylate Transporter 1
MDA	Malondialdehyde
MGBA	Microbiota–gut–brain axis
MRI	Magnetic resonance imaging
mTOR	Mechanistic Target of Rapamycin
n-3 PUFA	Omega-3 polyunsaturated fatty acids
NAA	N-acetylaspartate
NAC	N-acetil cysteine
NCLs	Neuronal ceroid lipofuscinoses
NF-κB	Nuclear factor kappa-light chain enhancer of activated B cells
Nrf2/HO-1	E2-related factor/haem oxygenase 1
NRF2	Nuclear factor erythroid 2
O_2_^−^	Superoxide anion
PD	Parkinson’s disease
PINK1	PTEN- induced kinase 1
PPARγ	Peroxisome Proliferator-Activated Receptor gamma
Prdx3	Peroxiredoxin 3
PTEN	Phosphatase and Tensin Homolog
PTZ	Pentylenetetrazol
PWE	Patients suffering from epilepsy
Q3G	Quercetin-3-O-glucuronide
RNS	Reactive nitrogen species
ROS	Reactive oxygen species
SCFAs	Short-chain fatty acids
SE	Status epilepticus
SIRT1	Silent information regulator 1
SLC2A1	Solute Carrier Family 2 Member 1
SLC5A8	Solute Carrier Family 5 Member 8
SMCT1	Sodium-coupled Monocarboxylate Transporter 1
TAC	Total antioxidant capacity
TDO	Tryptophan 2,3-dioxygenase
TGR5	Takeda G-protein-coupled receptor 5
TLE	Temporal lobe epilepsy
TMEV	Theiler’s murine encephalomyelitis virus
TNF-α	Tumor necrosis factor-alpha
TOS	Total oxidative stress
TPH1	Tryptophan hydroxylase 1
Trx2	Thioredoxin reductase 2
VNS	Vagus nerve stimulation
WAG/Rij	Wistar Albino Glaxo from Rijswijk
ZO-1	Zona occludes 1

## References

[1] Marchesi J.R., Adams D.H., Fava F., Hermes G.D., Hirschfield G.M., Hold G., Quraishi M.N., Kinross J., Smidt H., Tuohy K.M. (2016). The gut microbiota and host health: A new clinical frontier. Gut.

[2] John H.T., Thomas T.C., Chukwuebuka E.C., Ali A.B., Anass R., Tefera Y.Y., Babu B., Negrut N., Ferician A., Marian P. (2025). The Microbiota–Human Health Axis. Microorganisms.

[3] Liepke C., Adermann K., Raida M., Mägert H.J., Forssmann W.G., Zucht H.D. (2002). Human milk provides peptides highly stimulating the growth of bifidobacteria. Eur. J. Biochem..

[4] Aureli P., Capurso L., Castellazzi A.M., Clerici M., Giovannini M., Morelli L., Poli A., Pregliasco F., Salvini F., Zuccotti G.V. (2011). Probiotics and health: An evidence-based review. Pharmacol. Res..

[5] Bengmark S. (2013). Gut microbiota, immune development and function. Pharmacol. Res..

[6] Koga Y. (2022). Microbiota in the stomach and application of probiotics to gastroduodenal diseases. World J. Gastroenterol..

[7] Capurso L. (2016). Il microbiota intestinale [First part: The intestinal microbiota]. Recenti Progress. Med..

[8] Liu Y., Ma J., Zhu B., Liu F., Qin S., Lv N., Feng Y., Wang S., Yang H. (2023). A health-promoting role of exclusive breastfeeding on infants through restoring delivery mode-induced gut microbiota perturbations. Front. Microbiol..

[9] Shafik A.N., Fahim V.F., Iskander F.A., Elsayegh H.A., Serag H., Sallam H.S. (2025). The Role of the Gut Microbiome Dysbiosis in Metabolic Dysfunction: A Mini Review. Healthcare.

[10] Xu J., Lu Y. (2025). The microbiota-gut-brain axis and central nervous system diseases: From mechanisms of pathogenesis to therapeutic strategies. Front. Microbiol..

[11] Carabotti M., Scirocco A., Maselli M.A., Severi C. (2015). The gut-brain axis: Interactions between enteric microbiota, central and enteric nervous systems. Ann. Gastroenterol..

[12] Tsigos C., Chrousos G.P. (2002). Hypothalamic-pituitary-adrenal axis, neuroendocrine factors and stress. J. Psychosom. Res..

[13] Sharan P., Vellapandian C. (2024). Hypothalamic-Pituitary-Adrenal (HPA) Axis: Unveiling intial Mechanisms Involved in Stress-Induced Alzheimer’s Disease and Depression. Cureus.

[14] Gershon R.C., Cella D., Fox N.A., Havlik R.J., Hendrie H.C., Wagster M.V. (2010). Assessment of neurological and behavioural function: The NIH Toolbox. Lancet Neurol..

[15] Khlevner J., Park Y., Margolis K.G. (2018). Brain-Gut Axis: Clinical Implications. Gastroenterol. Clin. N. Am..

[16] Craig A.D. (2003). Interoception: The sense of the physiological condition of the body. Curr. Opin. Neurobiol..

[17] Shaffer C., Barrett L.F., Quigley K.S. (2023). Signal processing in the vagus nerve: Hypotheses based on new genetic and anatomical evidence. Biol. Psychol..

[18] Mayer E.A. (2011). Gut feelings: The emerging biology of gut-brain communication. Nat. Rev. Neurosci..

[19] Van Oudenhove L., Crowell M.D., Drossman D.A., Halpert A.D., Keefer L., Lackner J.M., Murphy T.B., Naliboff B.D., Levy R.L. (2016). Biopsychosocial Aspects of Functional Gastrointestinal Disorders: How Central and Environmental Processes Contribute to the Development and Expression of Functional Gastrointestinal Disorders. Gastroenterology.

[20] Craig A.D. (2002). How do you feel? Interoception: The sense of the physiological condition of the body. Nat. Rev. Neurosci..

[21] Jarrard L.E. (1993). On the role of the hippocampus in learning and memory in the rat. Behav. Neural Biol..

[22] Mello A.F., Mello M.F., Carpenter L.L., Price L.H. (2003). Update on stress and depression: The role of the hypothalamic-pituitary-adrenal (HPA) axis. Braz. J. Psychiatry.

[23] Stoodley C.J., Schmahmann J.D. (2010). Evidence for topographic organization in the cerebellum of motor control versus cognitive and affective processing. Cortex.

[24] Wang Q., Guo M., Liu Y., Xu M., Shi L., Li X., Zhao J., Zhang H., Wang G., Chen W. (2022). *Bifidobacterium breve* and *Bifidobacterium longum* Attenuate Choline-Induced Plasma Trimethylamine N-Oxide Production by Modulating Gut Microbiota in Mice. Nutrients.

[25] Maukonen J., Saarela M. (2015). Human gut microbiota: Does diet matter?. Proc. Nutr. Soc..

[26] Wang G., Yu Y., Wang Y.Z., Wang J.J., Guan R., Sun Y., Shi F., Gao J., Fu X.L. (2019). Role of SCFAs in gut microbiome and glycolysis for colorectal cancer therapy. J. Cell Physiol..

[27] Dalile B., Van Oudenhove L., Vervliet B., Verbeke K. (2019). The role of short-chain fatty acids in microbiota-gut-brain communication. Nat. Rev. Gastroenterol. Hepatol..

[28] O’Riordan K.J., Collins M.K., Moloney G.M., Knox E.G., Aburto M.R., Fülling C., Morley S.J., Clarke G., Schellekens H., Cryan J.F. (2022). Short chain fatty acids: Microbial metabolites for gut-brain axis signalling. Mol. Cell Endocrinol..

[29] Vijay N., Morris M.E. (2014). Role of monocarboxylate transporters in drug delivery to the brain. Curr. Pharm. Des..

[30] Mitchell R.W., On N.H., Del Bigio M.R., Miller D.W., Hatch G.M. (2011). Fatty acid transport protein expression in human brain and potential role in fatty acid transport across human brain microvessel endothelial cells. J. Neurochem..

[31] Saresella M., Marventano I., Barone M., La Rosa F., Piancone F., Mendozzi L., d’Arma A., Rossi V., Pugnetti L., Roda G. (2020). Alterations in Circulating Fatty Acid Are Associated with Gut Microbiota Dysbiosis and Inflammation in Multiple Sclerosis. Front. Immunol..

[32] Stanley D., Moore R.J., Wong C.H.Y. (2018). An insight into intestinal mucosal microbiota disruption after stroke. Sci. Rep..

[33] Opeyemi O.M., Rogers M.B., Firek B.A., Janesko-Feldman K., Vagni V., Mullett S.J., Wendell S.G., Nelson B.P., New L.A., Mariño E. (2021). Sustained Dysbiosis and Decreased Fecal Short-Chain Fatty Acids after Traumatic Brain Injury and Impact on Neurologic Outcome. J. Neurotrauma.

[34] Liu J., Sun J., Wang F., Yu X., Ling Z., Li H., Zhang H., Jin J., Chen W., Pang M. (2015). Neuroprotective Effects of Clostridium butyricum against Vascular Dementia in Mice via Metabolic Butyrate. Biomed. Res. Int..

[35] Liao H., Li H., Bao H., Jiang L., Du J., Guo Y., Si Y. (2022). Short Chain Fatty Acids Protect the Cognitive Function of Sepsis Associated Encephalopathy Mice via GPR43. Front. Neurol..

[36] Zhang L., Wang Y., Xiayu X., Shi C., Chen W., Song N., Fu X., Zhou R., Xu Y.F., Huang L. (2017). Altered Gut Microbiota in a Mouse Model of Alzheimer’s Disease. J. Alzheimers Dis..

[37] Wallen Z.D., Appah M., Dean M.N., Sesler C.L., Factor S.A., Molho E., Zabetian C.P., Standaert D.G., Payami H. (2020). Characterizing dysbiosis of gut microbiome in PD: Evidence for overabundance of opportunistic pathogens. npj Park. Dis..

[38] Bosi A., Banfi D., Bistoletti M., Giaroni C., Baj A. (2020). Tryptophan Metabolites Along the Microbiota-Gut-Brain Axis: An Interkingdom Communication System Influencing the Gut in Health and Disease. Int. J. Tryptophan Res..

[39] Dehhaghi M., Kazemi Shariat Panahi H., Guillemin G.J. (2019). Microorganisms, Tryptophan Metabolism, and Kynurenine Pathway: A Complex Interconnected Loop Influencing Human Health Status. Int. J. Tryptophan Res..

[40] Badawy A.A. (2017). Tryptophan availability for kynurenine pathway metabolism across the life span: Control mechanisms and focus on aging, exercise, diet and nutritional supplements. Neuropharmacology.

[41] Richard D.M., Dawes M.A., Mathias C.W., Acheson A., Hill-Kapturczak N., Dougherty D.M. (2009). L-Tryptophan: Basic Metabolic Functions, Behavioral Research and Therapeutic Indications. Int. J. Tryptophan Res..

[42] Cryan J.F., O’Riordan K.J., Cowan C.S.M., Sandhu K.V., Bastiaanssen T.F.S., Boehme M., Codagnone M.G., Cussotto S., Fulling C., Golubeva A.V. (2019). The Microbiota-Gut-Brain Axis. Physiol. Rev..

[43] Bistoletti M., Bosi A., Banfi D., Giaroni C., Baj A. (2020). The microbiota-gut-brain axis: Focus on the fundamental communication pathways. Prog. Mol. Biol. Transl. Sci..

[44] Monteiro-Cardoso V.F., Corlianò M., Singaraja R.R. (2021). Bile Acids: A Communication Channel in the Gut-Brain Axis. Neuromolecular Med..

[45] Rinninella E., Cintoni M., Raoul P., Lopetuso L.R., Scaldaferri F., Pulcini G., Miggiano G.A.D., Gasbarrini A., Mele M.C. (2019). Food Components and Dietary Habits: Keys for a Healthy Gut Microbiota Composition. Nutrients.

[46] David L.A., Maurice C.F., Carmody R.N., Gootenberg D.B., Button J.E., Wolfe B.E., Ling A.V., Devlin A.S., Varma Y., Fischbach M.A. (2014). Diet rapidly and reproducibly alters the human gut microbiome. Nature.

[47] Nissen L., Casciano F., Gianotti A. (2020). Intestinal fermentation in vitro models to study food-induced gut microbiota shift: An updated review. FEMS Microbiol. Lett..

[48] Duncan S.H., Belenguer A., Holtrop G., Johnstone A.M., Flint H.J., Lobley G.E. (2007). Reduced dietary intake of carbohydrates by obese subjects results in decreased concentrations of butyrate and butyrate-producing bacteria in feces. Appl. Environ. Microbiol..

[49] Walker A.W., Ince J., Duncan S.H., Webster L.M., Holtrop G., Ze X., Brown D., Stares M.D., Scott P., Bergerat A. (2011). Dominant and diet-responsive groups of bacteria within the human colonic microbiota. ISME J..

[50] Schroeder B.O., Birchenough G.M.H., Ståhlman M., Arike L., Johansson M.E.V., Hansson G.C., Bäckhed F. (2018). Bifidobacteria or Fiber Protects against Diet-Induced Microbiota-Mediated Colonic Mucus Deterioration. Cell Host Microbe.

[51] Hill C., Guarner F., Reid G., Gibson G.R., Merenstein D.J., Pot B., Morelli L., Canani R.B., Flint H.J., Salminen S. (2014). Expert consensus document. The International Scientific Association for Probiotics and Prebiotics consensus statement on the scope and appropriate use of the term probiotic. Nat. Rev. Gastroenterol. Hepatol..

[52] Lee H.J., Lee K.E., Kim J.K., Kim D.H. (2019). Suppression of gut dysbiosis by Bifidobacterium longum alleviates cognitive decline in 5XFAD transgenic and aged mice. Sci. Rep..

[53] Han D., Li Z., Liu T., Yang N., Li Y., He J., Qian M., Kuang Z., Zhang W., Ni C. (2020). Prebiotics Regulation of Intestinal Microbiota Attenuates Cognitive Dysfunction Induced by Surgery Stimulation in APP/PS1 Mice. Aging Dis..

[54] Luo L., Luo J., Cai Y., Fu M., Li W., Shi L., Liu J., Dong R., Xu X., Tu L. (2022). Inulin-type fructans change the gut microbiota and prevent the development of diabetic nephropathy. Pharmacol. Res..

[55] Mohapatra L., Mishra D., Tripathi A.S., Parida S.K., Palei N.N. (2025). Illustrating the Pathogenesis and Therapeutic Approaches of Epilepsy by Targeting Angiogenesis, Inflammation, and Oxidative Stress. Neuroglia.

[56] Borowicz-Reutt K.K., Czuczwar S.J. (2020). Role of oxidative stress in epileptogenesis and potential implications for therapy. Pharmacol. Rep..

[57] Sarmast S.T., Abdullahi A.M., Jahan N. (2020). Current Classification of Seizures and Epilepsies: Scope, Limitations and Recommendations for Future Action. Cureus.

[58] Pack A.M. (2019). Epilepsy Overview and Revised Classification of Seizures and Epilepsies. Continuum.

[59] Specchio N., Wirrell E.C., Scheffer I.E., Nabbout R., Riney K., Samia P., Guerreiro M., Gwer S., Zuberi S.M., Wilmshurst J.M. (2022). International League Against Epilepsy classification and definition of epilepsy syndromes with onset in childhood: Position paper by the ILAE Task Force on Nosology and Definitions. Epilepsia.

[60] Berg A.T., Berkovic S.F., Brodie M.J., Buchhalter J., Cross J.H., van Emde Boas W., Engel J., French J., Glauser T.A., Mathern G.W. (2010). Revised terminology and concepts for organization of seizures and epilepsies: Report of the ILAE Commission on Classification and Terminology, 2005-2009. Epilepsia.

[61] Baulac S., Huberfeld G., Gourfinkel-An I., Mitropoulou G., Beranger A., Prud’homme J.F., Baulac M., Brice A., Bruzzone R., LeGuern E. (2001). First genetic evidence of GABA(A) receptor dysfunction in epilepsy: A mutation in the gamma2-subunit gene. Nat. Genet..

[62] Wallace R.H., Marini C., Petrou S., Harkin L.A., Bowser D.N., Panchal R.G., Williams D.A., Sutherland G.R., Mulley J.C., Scheffer I.E. (2001). Mutant GABA(A) receptor gamma2-subunit in childhood absence epilepsy and febrile seizures. Nat. Genet..

[63] Lachance-Touchette P., Brown P., Meloche C., Kinirons P., Lapointe L., Lacasse H., Lortie A., Carmant L., Bedford F., Bowie D. (2011). Novel α1 and γ2 GABAA receptor subunit mutations in families with idiopathic generalized epilepsy. Eur. J. Neurosci..

[64] Maljevic S., Krampfl K., Cobilanschi J., Tilgen N., Beyer S., Weber Y.G., Schlesinger F., Ursu D., Melzer W., Cossette P. (2006). A mutation in the GABA(A) receptor alpha(1)-subunit is associated with absence epilepsy. Ann. Neurol..

[65] Johannesen K., Marini C., Pfeffer S., Møller R.S., Dorn T., Niturad C.E., Gardella E., Weber Y., Søndergård M., Hjalgrim H. (2016). Phenotypic spectrum of GABRA1: From generalized epilepsies to severe epileptic encephalopathies. Neurology.

[66] Koch H., Weber Y.G. (2019). The glucose transporter type 1 (Glut1) syndromes. Epilepsy Behav..

[67] Hosseini P., Homam M., Hosseini P., Badriahmadi N., Shamsi A. (2015). The Causes of Secondary Epilepsy in Epileptic Patients Referred to Neurology Clinics of Mashhad Hospitals. Shiraz E-Medical J..

[68] Henshall D.C., Kobow K. (2015). Epigenetics and Epilepsy. Cold Spring Harb. Perspect. Med..

[69] Zhu Q., Wang L., Zhang Y., Zhao F.H., Luo J., Xiao Z., Chen G.J., Wang X.F. (2012). Increased expression of DNA methyltransferase 1 and 3a in human temporal lobe epilepsy. J. Mol. Neurosci..

[70] Kobow K., Kaspi A., Harikrishnan K.N., Kiese K., Ziemann M., Khurana I., Fritzsche I., Hauke J., Hahnen E., Coras R. (2013). Deep sequencing reveals increased DNA methylation in chronic rat epilepsy. Acta Neuropathol..

[71] Miller-Delaney S.F., Das S., Sano T., Jimenez-Mateos E.M., Bryan K., Buckley P.G., Stallings R.L., Henshall D.C. (2012). Differential DNA methylation patterns define status epilepticus and epileptic tolerance. J. Neurosci..

[72] Huang Y., Zhao F., Wang L., Yin H., Zhou C., Wang X. (2012). Increased expression of histone deacetylases 2 in temporal lobe epilepsy: A study of epileptic patients and rat models. Synapse.

[73] Tsankova N.M., Kumar A., Nestler E.J. (2004). Histone modifications at gene promoter regions in rat hippocampus after acute and chronic electroconvulsive seizures. J. Neurosci..

[74] Hu J., Mo J.L., Cheng X.L. (2022). Study on MiRNA Epigenetic Intervention for Epilepsy. J. Biosci. Med..

[75] Jimenez-Mateos E.M., Bray I., Sanz-Rodriguez A., Engel T., McKiernan R.C., Mouri G., Tanaka K., Sano T., Saugstad J.A., Simon R.P. (2011). miRNA Expression profile after status epilepticus and hippocampal neuroprotection by targeting miR-132. Am. J. Pathol..

[76] Qureshi I.A., Mehler M.F. (2010). The emerging role of epigenetics in stroke: II. RNA regulatory circuitry. Arch. Neurol..

[77] Kobow K., Blümcke I. (2011). The methylation hypothesis: Do epigenetic chromatin modifications play a role in epileptogenesis?. Epilepsia.

[78] Afshari M., Pirzad Jahromi G., Roghani M. (2025). Dual strategies for epilepsy management employing pharmacological and non-invasive brain stimulation approaches. Front. Neurol..

[79] Riva A., Golda A., Balagura G., Amadori E., Vari M.S., Piccolo G., Iacomino M., Lattanzi S., Salpietro V., Minetti C. (2021). New Trends and Most Promising Therapeutic Strategies for Epilepsy Treatment. Front. Neurol..

[80] de Jesús Aguirre-Vera G., Montufar L., Tejada-Pineda M.F., Gomez M.P.F., Alvarez-Pinzon A., Valerio J.E., Luna-Ceron E. (2025). Targeting Drug-Resistant Epilepsy: A Narrative Review of Five Novel Antiseizure Medications. Int. J. Transl. Med..

[81] Bercik P., Denou E., Collins J., Jackson W., Lu J., Jury J., Deng Y., Blennerhassett P., Macri J., McCoy K.D. (2011). The intestinal microbiota affect central levels of brain-derived neurotropic factor and behavior in mice. Gastroenterology.

[82] Fusco F., Perottoni S., Giordano C., Riva A., Iannone L.F., De Caro C., Russo E., Albani D., Striano P. (2022). The microbiota-gut-brain axis and epilepsy from a multidisciplinary perspective: Clinical evidence and technological solutions for improvement of in vitro preclinical models. Bioeng. Transl. Med..

[83] Austelle C.W., Cox S.S., Wills K.E., Badran B.W. (2024). Vagus nerve stimulation (VNS): Recent advances and future directions. Clin. Auton. Res..

[84] Attenello F.J., Wen T., Cen S.Y., Ng A., Kim-Tenser M., Sanossian N., Amar A.P., Mack W.J. (2015). Incidence of “never events” among weekend admissions versus weekday admissions to US hospitals: National analysis. BMJ.

[85] Liebregts T., Adam B., Bredack C., Röth A., Heinzel S., Lester S., Downie-Doyle S., Smith E., Drew P., Talley N.J. (2007). Immune activation in patients with irritable bowel syndrome. Gastroenterology.

[86] Collins S.M., Surette M., Bercik P. (2012). The interplay between the intestinal microbiota and the brain. Nat. Rev. Microbiol..

[87] Keightley P.D., Pinharanda A., Ness R.W., Simpson F., Dasmahapatra K.K., Mallet J., Davey J.W., Jiggins C.D. (2015). Estimation of the spontaneous mutation rate in *Heliconius melpomene*. Mol. Biol. Evol..

[88] Qin H.Y., Cheng C.W., Tang X.D., Bian Z.X. (2014). Impact of psychological stress on irritable bowel syndrome. World J. Gastroenterol..

[89] Schaper S.J., Stengel A. (2022). Emotional stress responsivity of patients with IBS—A systematic review. J. Psychosom. Res..

[90] Mayer E.A., Tillisch K., Gupta A. (2015). Gut/brain axis and the microbiota. J. Clin. Invest..

[91] Berthoud H.R., Morrison C. (2008). The brain, appetite, and obesity. Annu. Rev. Psychol..

[92] Cano-López I., Hidalgo V., Hampel K.G., Garcés M., Salvador A., González-Bono E., Villanueva V. (2019). Cortisol and trait anxiety as relevant factors involved in memory performance in people with drug-resistant epilepsy. Epilepsy Behav..

[93] Chen T.-S., Huang T.-H., Lai M.-C., Huang C.-W. (2023). The Role of Glutamate Receptors in Epilepsy. Biomedicines.

[94] Reddy D.S., Rogawski M.A. (2002). Stress-induced deoxycorticosterone-derived neurosteroids modulate GABA(A) receptor function and seizure susceptibility. J. Neurosci..

[95] Werner F.M., Coveñas R. (2017). Classical neurotransmitters and neuropeptides involved in generalized epilepsy in a multi-neurotransmitter system: How to improve the antiepileptic effect?. Epilepsy Behav..

[96] Galland L. (2014). The gut microbiome and the brain. J. Med. Food..

[97] McDonald J.A.K., Mullish B.H., Pechlivanis A., Liu Z., Brignardello J., Kao D., Holmes E., Li J.V., Clarke T.B., Thursz M.R. (2018). Inhibiting Growth of Clostridioides difficile by Restoring Valerate, Produced by the Intestinal Microbiota. Gastroenterology.

[98] Olson C.A., Vuong H.E., Yano J.M., Liang Q.Y., Nusbaum D.J., Hsiao E.Y. (2018). The Gut Microbiota Mediates the Anti-Seizure Effects of the Ketogenic Diet. Cell.

[99] Yano J.M., Yu K., Donaldson G.P., Shastri G.G., Ann P., Ma L., Nagler C.R., Ismagilov R.F., Mazmanian S.K., Hsiao E.Y. (2015). Indigenous bacteria from the gut microbiota regulate host serotonin biosynthesis. Cell.

[100] Lu S., Wei F., Li G. (2021). The evolution of the concept of stress and the framework of the stress system. Cell Stress..

[101] Petrucci A.N., Joyal K.G., Purnell B.S., Buchanan G.F. (2020). Serotonin and sudden unexpected death in epilepsy. Exp. Neurol..

[102] Ferrier S., Pressey R., Barrett T. (2000). A new predictor of the irreplaceability of areas for achieving a conservation goal, its application to real-world planning, and a research agenda for further refinement. Biol. Conserv..

[103] Gomes J.P., Bruno W.J., Nunes A., Santos N., Florindo C., Borrego M.J., Dean D. (2007). Evolution of Chlamydia trachomatis diversity occurs by widespread interstrain recombination involving hotspots. Genome Res..

[104] den Besten G., van Eunen K., Groen A.K., Venema K., Reijngoud D.J., Bakker B.M. (2013). The role of short-chain fatty acids in the interplay between diet, gut microbiota, and host energy metabolism. J. Lipid Res..

[105] De Caro C., Iannone L.F., Citraro R., Striano P., De Sarro G., Constanti A., Cryan J.F., Russo E. (2019). Can we ‘seize’ the gut microbiota to treat epilepsy?. Neurosci. Biobehav. Rev..

[106] Xie G., Zhou Q., Qiu C.Z., Dai W.K., Wang H.P., Li Y.H., Liao J.X., Lu X.G., Lin S.F., Ye J.H. (2017). Ketogenic diet poses a significant effect on imbalanced gut microbiota in infants with refractory epilepsy. World J. Gastroenterol..

[107] Lee D.A., Kim B.J., Lee H.J., Kim S.E., Park K.M. (2020). Network characteristics of genetic generalized epilepsy: Are the syndromes distinct?. Seizure.

[108] Şafak B., Altunan B., Topçu B., Eren Topkaya A. (2020). The gut microbiome in epilepsy. Microb. Pathog..

[109] Medel-Matus J.S., Lagishetty V., Santana-Gomez C., Shin D., Mowrey W., Staba R.J., Galanopoulou A.S., Sankar R., Jacobs J.P., Mazarati A.M. (2022). Susceptibility to epilepsy after traumatic brain injury is associated with preexistent gut microbiome profile. Epilepsia.

[110] Li Z., Cao W., Sun H., Wang X., Li S., Ran X., Zhang H. (2022). Potential clinical and biochemical markers for the prediction of drug-resistant epilepsy: A literature review. Neurobiol. Dis..

[111] Cheng C.W., Biton M., Haber A.L., Gunduz N., Eng G., Gaynor L.T., Tripathi S., Calibasi-Kocal G., Rickelt S., Butty V.L. (2019). Ketone Body Signaling Mediates Intestinal Stem Cell Homeostasis and Adaptation to Diet. Cell.

[112] Dahlin M., Wheelock C.E., Prast-Nielsen S. (2024). Association between seizure reduction during ketogenic diet treatment of epilepsy and changes in circulatory metabolites and gut microbiota composition. EBioMedicine.

[113] Iannone L.F., Preda A., Blottière H.M., Clarke G., Albani D., Belcastro V., Carotenuto M., Cattaneo A., Citraro R., Ferraris C. (2019). Microbiota-gut brain axis involvement in neuropsychiatric disorders. Expert. Rev. Neurother..

[114] Landgrave-Gómez J., Mercado-Gómez O., Guevara-Guzmán R. (2015). Epigenetic mechanisms in neurological and neurodegenerative diseases. Front. Cell Neurosci..

[115] Morris G., Fernandes B.S., Puri B.K., Walker A.J., Carvalho A.F., Berk M. (2018). Leaky brain in neurological and psychiatric disorders: Drivers and consequences. Aust. N. Z. J. Psychiatry.

[116] Simeonova D., Ivanovska M., Murdjeva M., Carvalho A.F., Maes M. (2018). Recognizing the Leaky Gut as a Trans-diagnostic Target for Neuroimmune Disorders Using Clinical Chemistry and Molecular Immunology Assays. Curr. Top. Med. Chem..

[117] Cenit M.C., Sanz Y., Codoñer-Franch P. (2017). Influence of gut microbiota on neuropsychiatric disorders. World J. Gastroenterol..

[118] Swissa E., Serlin Y., Vazana U., Prager O., Friedman A. (2019). Blood-brain barrier dysfunction in status epileptics: Mechanisms and role in epileptogenesis. Epilepsy Behav..

[119] Sun W., Wang Y., Xiao B., Luo Z. (2025). From the different pathogenesis of epileptogenesis: Vitamins as an adjunctive treatment for epilepsy. Acta Epileptol..

[120] Castelli S., Carinci E., Baldelli S. (2025). Oxidative Stress in Neurodegenerative Disorders: A Key Driver in Impairing Skeletal Muscle Health. Int. J. Mol. Sci..

[121] Kim G.H., Kim J.E., Rhie S.J., Yoon S. (2015). The Role of Oxidative Stress in Neurodegenerative Diseases. Exp. Neurobiol..

[122] Lamptey R.N.L., Chaulagain B., Trivedi R., Gothwal A., Layek B., Singh J. (2022). A Review of the Common Neurodegenerative Disorders: Current Therapeutic Approaches and the Potential Role of Nanotherapeutics. Int. J. Mol. Sci..

[123] Han J., Wang Y., Wei P., Lu D., Shan Y. (2024). Unveiling the hidden connection: The blood-brain barrier’s role in epilepsy. Front. Neurol..

[124] Cardenas N., Coballase-Urrutia E., Perez-Cruz C., Montesinos-Correa H., Rivera L., Sampieri A., Carmona-Aparicio L. (2014). Relevance of the Glutathione System in Temporal Lobe Epilepsy: Evidence in Human and Experimental Models. Oxidative Med. Cell. Longev..

[125] Giustarini D., Milzani A., Dalle-Donne I., Rossi R. (2023). How to Increase Cellular Glutathione. Antioxidant.

[126] Milder J., Patel M. (2012). Modulation of oxidative stress and mitochondrial function by the ketogenic diet. Epilepsy Res..

[127] Mueller S.G., Trabesinger A.H., Boesiger P., Wieser H.G. (2001). Brain glutathione levels in patients with epilepsy measured by in vivo (1)H-MRS. Neurology.

[128] Strine T.W., Kobau R., Chapman D.P., Thurman D.J., Price P., Balluz L.S. (2005). Psychological distress, comorbidities, and health behaviors among U.S. adults with seizures: Results from the 2002 National Health Interview Survey. Epilepsia.

[129] Pence S., Erkutlu I., Kurtul N., Bosnak M., Alptekin M., Tan U. (2009). Antiepileptogenic Effects of Glutathione Against Increased Brain ADA in PTZ-Induced Epilepsy. Int. J. Neurosci..

[130] Mu C., Nikpoor N., Tompkins T.A., Choudhary A., Wang M., Marks W.N., Rho J.M., Scantlebury M.H., Shearer J. (2022). Targeted gut microbiota manipulation attenuates seizures in a model of infantile spasms syndrome. JCI Insight.

[131] Napolitano A., Longo D., Lucignani M., Pasquini L., Rossi-Espagnet M.C., Lucignani G., Maiorana A., Elia D., De Liso P., Dionisi-Vici C. (2020). The Ketogenic Diet Increases In Vivo Glutathione Levels in Patients with Epilepsy. Metabolites.

[132] Pauletti A., Terrone G., Shekh-Ahmad T., Salamone A., Ravizza T., Rizzi M., Pastore A., Pascente R., Liang L.P., Villa B.R. (2019). Targeting oxidative stress improves disease outcomes in a rat model of acquired epilepsy. Brain.

[133] McElroy P.B., Liang L.P., Day B.J., Patel M. (2017). Scavenging reactive oxygen species inhibits status epilepticus-induced neuroinflammation. Exp. Neurol..

[134] Koh S., Dupuis N., Auvin S. (2020). Ketogenic diet and Neuroinflammation. Epilepsy Res..

[135] Bhuyan P., Patel D.C., Wilcox K.S., Patel M. (2015). Oxidative stress in murine Theiler’s virus-induced temporal lobe epilepsy. Exp. Neurol..

[136] Fabisiak T., Patel M. (2022). Crosstalk between neuroinflammation and oxidative stress in epilepsy. Front. Cell Dev. Biol..

[137] Groitl B., Jakob U. (2014). Thiol-based redox switches. Biochim. Biophys. Acta.

[138] Villasana-Salazar B., Vezzani A. (2023). Neuroinflammation microenvironment sharpens seizure circuit. Neurobiol. Dis..

[139] Morel J., Berenbaum F. (2004). Signal transduction pathways: New targets for treating rheumatoid arthritis. Jt. Bone Spine.

[140] El-ghoroury E.A., Raslan H.M., Badawy E.A., El-Saaid G.S., Agybi M.H., Siam I., Salem S.I. (2009). Malondialdehyde and coenzyme Q10 in platelets and serum in type 2 diabetes mellitus: Correlation with glycemic control. Blood Coagul. Fibrinolysis.

[141] Lamperti C., Naini A., Hirano M., De Vivo D.C., Bertini E., Servidei S., Valeriani M., Lynch D., Banwell B., Berg M. (2003). Cerebellar ataxia and coenzyme Q10 deficiency. Neurology.

[142] Simani L., Ryan F., Hashemifard S., Hooshmandi E., Madahi M., Sahraei Z., Rezaei O., Heydari K., Ramezani M. (2018). Serum Coenzyme Q10 Is Associated with Clinical Neurological Outcomes in Acute Stroke Patients. J. Mol. Neurosci..

[143] Chew G.T., Watts G.F. (2004). Coenzyme Q10 and diabetic endotheliopathy: Oxidative stress and the ‘recoupling hypothesis’. QJM.

[144] Cengiz M., Yüksel A., Seven M. (2000). The effects of carbamazepine and valproic acid on the erythrocyte glutathione, glutathione peroxidase, superoxide dismutase and serum lipid peroxidation in epileptic children. Pharmacol. Res..

[145] Yiş U., Seçkin E., Kurul S.H., Kuralay F., Dirik E. (2009). Effects of epilepsy and valproic acid on oxidant status in children with idiopathic epilepsy. Epilepsy Res..

[146] Tan D.X., Manchester L.C., Reiter R.J., Qi W., Kim S.J., El-Sokkary G.H. (1998). Melatonin protects hippocampal neurons in vivo against kainic acid-induced damage in mice. J. Neurosci. Res..

[147] Breier M.R., Lewis B., Shoemaker J.M., Light G.A., Swerdlow N.R. (2010). Sensory and sensorimotor gating-disruptive effects of apomorphine in Sprague Dawley and Long Evans rats. Behav. Brain Res..

[148] Rinninella E., Tohumcu E., Raoul P., Fiorani M., Cintoni M., Mele M.C., Cammarota G., Gasbarrini A., Ianiro G. (2023). The role of diet in shaping human gut microbiota. Best. Pract. Res. Clin. Gastroenterol..

[149] Zarnowska I.M. (2020). Therapeutic Use of the Ketogenic Diet in Refractory Epilepsy: What We Know and What Still Needs to Be Learned. Nutrients.

[150] Liu L., Qi W., Zhang N., Zhang J., Liu S., Wang H., Jiang L., Sun Y. (2025). Nutraceuticals for Gut–Brain Axis Health: A Novel Approach to Combat Malnutrition and Future Personalised Nutraceutical Interventions. Nutrients.

[151] Pittman Q.J. (2020). A gut feeling about the ketogenic diet in epilepsy. Epilepsy Res..

[152] Hallböök T., Sjölander A., Åmark P., Miranda M., Bjurulf B., Dahlin M. (2015). Effectiveness of the ketogenic diet used to treat resistant childhood epilepsy in Scandinavia. Eur. J. Paediatr Neurol..

[153] Zhang Y., Zhou S., Zhou Y., Yu L., Zhang L., Wang Y. (2018). Altered gut microbiome composition in children with refractory epilepsy after ketogenic diet. Epilepsy Res..

[154] Hertz L. (2013). The Glutamate-Glutamine (GABA) Cycle: Importance of Late Postnatal Development and Potential Reciprocal Interactions between Biosynthesis and Degradation. Front Endocrinol..

[155] Lindefeldt M., Eng A., Darban H., Bjerkner A., Zetterström C.K., Allander T., Andersson B., Borenstein E., Dahlin M., Prast-Nielsen S. (2019). The ketogenic diet influences taxonomic and functional composition of the gut microbiota in children with severe epilepsy. npj Biofilms Microbiomes.

[156] O’Callaghan A., van Sinderen D. (2016). Bifidobacteria and Their Role as Members of the Human Gut Microbiota. Front. Microbiol..

[157] Zhu H., Wang W., Li Y. (2024). The interplay between microbiota and brain-gut axis in epilepsy treatment. Front. Pharmacol..

[158] Gudan A., Skonieczna-Żydecka K., Palma J., Drozd A., Stachowska E. (2022). Effects of dietary components on intestinal short-chain fatty acids (SCFAs) synthesis in healthy adult persons following a ketogenic diet. Rocz. Panstw. Zakl. Hig..

[159] Freeman J.M., Vining E.P., Pillas D.J., Pyzik P.L., Casey J.C., Kelly L.M. (1998). The efficacy of the ketogenic diet-1998: A prospective evaluation of intervention in 150 children. Pediatrics.

[160] Lefevre F., Aronson N. (2000). Ketogenic diet for the treatment of refractory epilepsy in children: A systematic review of efficacy. Pediatrics.

[161] Sampaio L.P. (2016). Ketogenic diet for epilepsy treatment. Arq. Neuro-Psiquiatr..

[162] Warren E.C., Walker M.C., Williams R.S.B. (2018). All You Need Is Fats-for Seizure Control: Using Amoeba to Advance Epilepsy Research. Front. Cell Neurosci..

[163] Ruan Y., Chen L., She D., Chung Y., Ge L., Han L. (2022). Ketogenic diet for epilepsy: An overview of systematic review and meta-analysis. Eur. J. Clin. Nutr..

[164] Boison D. (2017). New insights into the mechanisms of the ketogenic diet. Curr. Opin. Neurol..

[165] Chang P., Zuckermann A.M., Williams S., Close A.J., Cano-Jaimez M., McEvoy J.P., Spencer J., Walker M.C., Williams R.S. (2015). Seizure control by derivatives of medium chain fatty acids associated with the ketogenic diet show novel branching-point structure for enhanced potency. J. Pharmacol. Exp. Ther..

[166] Khabbush A., Orford M., Tsai Y.C., Rutherford T., O’Donnell M., Eaton S., Heales S.J.R. (2017). Neuronal decanoic acid oxidation is markedly lower than that of octanoic acid: A mechanistic insight into the medium-chain triglyceride ketogenic diet. Epilepsia.

[167] Mhanna A., Mhanna M., Beran A., Al-Chalabi M., Aladamat N., Mahfooz N. (2022). Modified Atkins diet versus ketogenic diet in children with drug-resistant epilepsy: A meta-analysis of comparative studies. Clin. Nutr. ESPEN.

[168] Devi N., Madaan P., Kandoth N., Bansal D., Sahu J.K. (2023). Efficacy and Safety of Dietary Therapies for Childhood Drug-Resistant Epilepsy: A Systematic Review and Network Meta-analysis. JAMA Pediatr..

[169] Chang P., Augustin K., Boddum K., Williams S., Sun M., Terschak J.A., Hardege J.D., Chen P.E., Walker M.C., Williams R.S. (2016). Seizure control by decanoic acid through direct AMPA receptor inhibition. Brain.

[170] Skrobas U., Duda P., Bryliński Ł., Drożak P., Pelczar M., Rejdak K. (2022). Ketogenic Diets in the Management of Lennox-Gastaut Syndrome—Review of Literature. Nutrients.

[171] Mahmoud S.H., Ho-Huang E., Buhler J. (2019). Systematic review of ketogenic diet use in adult patients with status epilepticus. Epilepsia Open.

[172] Groesbeck D.K., Bluml R.M., Kossoff E.H. (2006). Long-term use of the ketogenic diet in the treatment of epilepsy. Dev. Med. Child. Neurol..

[173] He F., Qiu J., Li H., Guo H., Wang S., Ding Y., Xu S., Wang Z., Feng J., Zhang P. (2022). Efficacy of the ketogenic diet in Chinese adults versus children with drug-resistant epilepsy: A pilot study. Epilepsy Behav..

[174] Iannone L.F., Gómez-Eguílaz M., De Caro C. (2022). Gut microbiota manipulation as an epilepsy treatment. Neurobiol. Dis..

[175] Sharma P., Agrawal A. (2022). Does modern research validate the ancient wisdom of gut flora and brain connection? A literature review of gut dysbiosis in neurological and neurosurgical disorders over the last decade. Neurosurg. Rev..

[176] Tremlett H., Bauer K.C., Appel-Cresswell S., Finlay B.B., Waubant E. (2017). The gut microbiome in human neurological disease: A review. Ann. Neurol..

[177] Riva A., Pozzati E., Grasso M., De Caro C., Russo E., Verrotti A., Striano P. (2022). Targeting the MGBA with -biotics in epilepsy: New insights from preclinical and clinical studies. Neurobiol. Dis..

[178] Arulsamy A., Shaikh M.F. (2022). Epilepsy-associated comorbidities among adults: A plausible therapeutic role of gut microbiota. Neurobiol. Dis..

[179] Chen C., Wang G.Q., Li D.D., Zhang F. (2025). Microbiota-gut-brain axis in neurodegenerative diseases: Molecular mechanisms and therapeutic targets. Mol. Biomed..

[180] Qin L., Wu X., Block M.L., Liu Y., Breese G.R., Hong J.S., Knapp D.J., Crews F.T. (2007). Systemic LPS causes chronic neuroinflammation and progressive neurodegeneration. Glia.

[181] Lee H., Ryu J., Nam E., Chung S.J., Yeo Y., Park D.W., Park T.S., Moon J.Y., Kim T.H., Sohn J.W. (2019). Increased mortality in patients with corticosteroid-dependent asthma: A nationwide population-based study. Eur. Respir. J..

[182] Ou Z., Deng L., Lu Z., Wu F., Liu W., Huang D., Peng Y. (2020). Protective effects of Akkermansia muciniphila on cognitive deficits and amyloid pathology in a mouse model of Alzheimer’s disease. Nutr. Diabetes.

[183] Chen L.-H., Wang M.-F., Chang C.-C., Huang S.-Y., Pan C.-H., Yeh Y.-T., Huang C.-H., Chan C.-H., Huang H.-Y. (2021). *Lacticaseibacillus paracasei* PS23 Effectively Modulates Gut Microbiota Composition and Improves Gastrointestinal Function in Aged SAMP8 Mice. Nutrients.

[184] Hao X., Ding N., Zhang Y., Yang Y., Zhao Y., Zhao J., Li Y., Li Z. (2022). Benign regulation of the gut microbiota: The possible mechanism through which the beneficial effects of manual acupuncture on cognitive ability and intestinal mucosal barrier function occur in APP/PS1 mice. Front Neurosci..

[185] Jiang H., Wang X., Zhou W., Huang Z., Zhang W. (2025). Gut microbiota-derived short-chain fatty acids mediate the antifibrotic effects of traditional Chinese medicine in diabetic nephropathy. Front Endocrinol.

[186] Kim S., Park S., Choi T.G., Kim S.S. (2022). Role of Short Chain Fatty Acids in Epilepsy and Potential Benefits of Probiotics and Prebiotics: Targeting “Health” of Epileptic Patients. Nutrients.

[187] Szczuko M., Duliban G., Drozd A., Sochaczewska D., Pokorska-Niewiada K., Ziętek M. (2024). The Association of Short-Chain Fatty Acids with the Occurrence of Gastrointestinal Symptoms in Infants. Int. J. Mol. Sci..

[188] Wen X., Zhao H., Wang L., Wang L., Du G., Guan W., Liu J., Cao X., Jiang X., Tian J. (2020). Nobiletin Attenuates DSS-Induced Intestinal Barrier Damage through the HNF4α-Claudin-7 Signaling Pathway. J. Agric. Food Chem..

[189] Li X., Yang H., Yan J., Wang X., Yuan Y., Li X. (2019). Seizure control by low-intensity ultrasound in mice with temporal lobe epilepsy. Epilepsy Res..

[190] Hoyles L., Snelling T., Umlai U.K., Nicholson J.K., Carding S.R., Glen R.C., McArthur S. (2018). Microbiome-host systems interactions: Protective effects of propionate upon the blood-brain barrier. Microbiome.

[191] Shi Y., Liu S., Wang J., Li C., Zhang J. (2021). Stigma experienced by patients with epilepsy: A systematic review and meta-synthesis of qualitative studies. Epilepsy Behav..

[192] Wang S., Wang I.Z., Bulacio J.C., Mosher J.C., Gonzalez-Martinez J., Alexopoulos A.V., Najm I.M., So N.K. (2013). Ripple classification helps to localize the seizure-onset zone in neocortical epilepsy. Epilepsia.

[193] De Caro C., Leo A., Nesci V., Ghelardini C., di Cesare Mannelli L., Striano P., Avagliano C., Calignano A., Mainardi P., Constanti A. (2019). Intestinal inflammation increases convulsant activity and reduces antiepileptic drug efficacy in a mouse model of epilepsy. Sci. Rep..

[194] Huang S.-Y., Chen L.-H., Wang M.-F., Hsu C.-C., Chan C.-H., Li J.-X., Huang H.-Y. (2018). *Lactobacillus paracasei* PS23 Delays Progression of Age-Related Cognitive Decline in Senescence Accelerated Mouse Prone 8 (SAMP8) Mice. Nutrients.

[195] Zhai J., Wang C., Jin L., Liu F., Xiao Y., Gu H., Liu M., Chen Y. (2024). Gut Microbiota Metabolites Mediate Bax to Reduce Neuronal Apoptosis via cGAS/STING Axis in Epilepsy. Mol. Neurobiol..

[196] Decout A., Katz J.D., Venkatraman S., Ablasser A. (2021). The cGAS-STING pathway as a therapeutic target in inflammatory diseases. Nat. Rev. Immunol..

[197] Ciltas A.C., Toy C.E., Güneş H., Yaprak M. (2023). Effects of probiotics on GABA/glutamate and oxidative stress in PTZ- induced acute seizure model in rats. Epilepsy Res..

[198] Gouveia T.L., Vieira de Sousa P.V., de Almeida S.S., Nejm M.B., Vieira de Brito J.M., Cysneiros R.M., de Brito M.V., Salu B.R., Oliva M.L., Scorza F.A. (2015). High serum levels of proinflammatory markers during epileptogenesis. Can omega-3 fatty acid administration reduce this process?. Epilepsy Behav..

[199] Rao M.L., Stefan H., Scheid C., Kuttler A.D., Fröscher W. (1993). Serum amino acids, liver status, and antiepileptic drug therapy in epilepsy. Epilepsia.

[200] Rojas A., Chen D., Ganesh T., Varvel N.H., Dingledine R. (2019). The COX-2/prostanoid signaling cascades in seizure disorders. Expert. Opin. Ther. Targets.

[201] Cândido F.G., Valente F.X., Grześkowiak Ł.M., Moreira A.P.B., Rocha D.M.U.P., Alfenas R.C.G. (2018). Impact of dietary fat on gut microbiota and low-grade systemic inflammation: Mechanisms and clinical implications on obesity. Int. J. Food Sci. Nutr..

[202] Lauritzen I., Blondeau N., Heurteaux C., Widmann C., Romey G., Lazdunski M. (2000). Polyunsaturated fatty acids are potent neuroprotectors. EMBO J..

[203] Verrotti A., Lattanzi S., Brigo F., Zaccara G. (2020). Pharmacodynamic interactions of antiepileptic drugs: From bench to clinical practice. Epilepsy Behav..

[204] Endriastuti N., Suryoputri M., Ilma D., Purwonugroho T. (2022). The potential interaction between antiepileptic drugs and nutraceuticals used in pediatrics with epilepsy. Acta Pharm. Indones. Acta Pharm Indo.

[205] Junges C., Machado T.D., Nunes Filho P.R.S., Riesgo R., Mello E.D. (2020). Vitamin D deficiency in pediatric patients using antiepileptic drugs: Systematic review with meta-analysis. J. Pediatr..

[206] Christiansen C., Rodbro P., Sjö O. (1974). “Anticonvulsant action” of vitamin D in epileptic patients? A controlled pilot study. Br. Med. J..

[207] Holló A., Clemens Z., Kamondi A., Lakatos P., Szűcs A. (2012). Correction of vitamin D deficiency improves seizure control in epilepsy: A pilot study. Epilepsy Behav..

[208] Upaganlawar A.B., Wankhede N.L., Kale M.B., Umare M.D., Sehgal A., Singh S., Bhatia S., Al-Harrasi A., Najda A., Nurzyńska-Wierdak R. (2021). Interweaving epilepsy and neurodegeneration: Vitamin E as a treatment approach. Biomed. Pharmacother..

[209] Bahalul-Yarchi S., Hartman F., Ben Zaken K., Sawaid I.O., Segev L., Mesfin S., Frankel P., Ezzy R., Samson A.O. (2025). Drugs and Nutrients in Epilepsy: Vitamin B6 and the Ketogenic Diet. Nutrients.

[210] Tavan M., Hanachi P., de la Luz Cádiz-Gurrea M., Segura Carretero A., Mirjalili M.H. (2024). Natural Phenolic Compounds with Neuroprotective Effects. Neurochem. Res..

[211] Hu B., Ouyang Y., Zhao T., Wang Z., Yan Q., Qian Q., Wang W., Wang S. (2024). Antioxidant Hydrogels: Antioxidant Mechanisms, Design Strategies, and Applications in the Treatment of Oxidative Stress-Related Diseases. Adv. Healthc. Mater..

[212] Yahia I., Baccouri O., Jalouli M., Boujelbene N., Rahman M.D.A., Harrath A.H., Zidi I. (2024). The small phytomolecule resveratrol: A promising role in boosting tumor cell chemosensitivity. Pharmacia.

[213] Bastos de Araújo D., Gurgel do Amaral A.L., Maia da Fonseca S., Rodrigues de Souza K., Santos da Paz A.P., Jóia de Mello V., Barbosa G.B., Otake Hamoy M.K., Hamoy M. (2023). Lippia origanoides essential oil possesses anticonvulsant effect in pentylenetetrazol-induced seizures in rats: A behavioral, electroencephalographic, and electromyographic study. Front. Pharmacol..

[214] Xiaoyu C., Hongzhen Z., Nan P., Tengwei G., Yanan G., Yan G., Haiyan L., Li M., Haiya W., Yujun W. (2024). Benzyl isothiocyanate ameliorates cognitive function in mice of chronic temporal lobe epilepsy. Front. Neurol..

[215] Li C., Wang N., Zheng G., Yang L. (2021). Oral Administration of Resveratrol-Selenium-Peptide Nanocomposites Alleviates Alzheimer’s Disease-like Pathogenesis by Inhibiting Aβ Aggregation and Regulating Gut Microbiota. ACS Appl. Mater. Interfaces.

[216] Xu Y., Xie M., Xue J., Xiang L., Li Y., Xiao J., Xiao G., Wang H.L. (2020). EGCG ameliorates neuronal and behavioral defects by remodeling gut microbiota and TotM expression in Drosophila models of Parkinson’s disease. FASEB J..

[217] Shen L., Liu L., Li X.Y., Ji H.F. (2019). Regulation of gut microbiota in Alzheimer’s disease mice by silibinin and silymarin and their pharmacological implications. Appl. Microbiol. Biotechnol..

[218] Mugundhan V., Arthanari A., Parthasarathy P.R. (2024). Protective Effect of Ferulic Acid on Acetylcholinesterase and Amyloid Beta Peptide Plaque Formation in Alzheimer’s Disease: An In Vitro Study. Cureus.

[219] Choi G.Y., Kim H.B., Hwang E.S., Park H.S., Cho J.M., Ham Y.K., Kim J.H., Mun M.K., Maeng S., Park J.H. (2023). Naringin enhances long-term potentiation and recovers learning and memory deficits of amyloid-beta induced Alzheimer’s disease-like behavioral rat model. Neurotoxicology.

[220] Cui C., Han Y., Li H., Yu H., Zhang B., Li G. (2022). Curcumin-driven reprogramming of the gut microbiota and metabolome ameliorates motor deficits and neuroinflammation in a mouse model of Parkinson’s disease. Front. Cell Infect. Microbiol..

[221] Xu M., Huang H., Mo X., Zhu Y., Chen X., Li X., Peng X., Xu Z., Chen L., Rong S. (2021). Quercetin-3-O-Glucuronide Alleviates Cognitive Deficit and Toxicity in Aβ1-42 -Induced AD-Like Mice and SH-SY5Y Cells. Mol. Nutr. Food Res..

[222] Shukitt-Hale B., Thangthaeng N., Miller M.G., Poulose S.M., Carey A.N., Fisher D.R. (2019). Blueberries Improve Neuroinflammation and Cognition differentially Depending on Individual Cognitive baseline Status. J. Gerontol. A Biol. Sci. Med. Sci..

[223] Johnston G.A.R., Beart P.M. (2024). Milestone review: GABA, from chemistry, conformations, ionotropic receptors, modulators, epilepsy, flavonoids, and stress to neuro-nutraceuticals. J. Neurochem..

[224] Di Meo F., Margarucci S., Galderisi U., Crispi S., Peluso G. (2019). Curcumin, Gut Microbiota, and Neuroprotection. Nutrients.

[225] Chung J.Y., Jeong J.H., Song J. (2020). Resveratrol Modulates the Gut-Brain Axis: Focus on Glucagon-Like Peptide-1, 5-HT, and Gut Microbiota. Front. Aging Neurosci..

[226] Wang Y., Chen S., Xu Z., Chen S., Yao W., Gao X. (2018). GLP-1 receptor agonists downregulate aberrant GnT-III expression in Alzheimer’s disease models through the Akt/GSK-3β/β-catenin signaling. Neuropharmacology.

[227] Charrière K., Schneider V., Perrignon-Sommet M., Lizard G., Benani A., Jacquin-Piques A., Vejux A. (2024). Exploring the Role of Apigenin in Neuroinflammation: Insights and Implications. Int. J. Mol. Sci..

[228] Zhong H., Xu J., Yang M., Hussain M., Liu X., Feng F., Guan R. (2023). Protective Effect of Anthocyanins against Neurodegenerative Diseases through the Microbial-Intestinal-Brain Axis: A Critical Review. Nutrients.

[229] Ticinesi A., Tana C., Nouvenne A., Prati B., Lauretani F., Meschi T. (2018). Gut microbiota, cognitive frailty and dementia in older individuals: A systematic review. Clin. Interv. Aging.

[230] Qu X., Zhang L., Wang L. (2023). Pterostilbene as a Therapeutic Alternative for Central Nervous System Disorders: A Review of the Current Status and Perspectives. J. Agric. Food Chem..

[231] Pontifex M.G., Malik M.M.A.H., Connell E., Müller M., Vauzour D. (2021). Citrus Polyphenols in Brain Health and Disease: Current Perspectives. Front. Neurosci..

[232] Rauf A., Abu-Izneid T., Imran M., Hemeg H.A., Bashir K., Aljohani A.S.M., Aljohani M.S.M., Alhumaydhi F.A., Khan I.N., Bin Emran T. (2023). Therapeutic Potential and Molecular Mechanisms of the Multitargeted Flavonoid Fisetin. Curr. Top. Med. Chem..

[233] Dogra S.K., Kajla V., Garg S., Singh H., Kumar D., Puri G., Kaur M., Kumar A., Bilandi A. (2023). Genistein as a neuroprotective agent: A comprehensive review of its potential in neurodegenerative diseases. J. Pharma Insights Res..

[234] Madireddy S., Madireddy S. (2023). Therapeutic Strategies to Ameliorate Neuronal Damage in Epilepsy by Regulating Oxidative Stress, Mitochondrial Dysfunction, and Neuroinflammation. Brain Sci..

[235] Jalouli M., Rahman M.A., Biswas P., Rahman H., Harrath A.H., Lee I.S., Kang S., Choi J., Park M.N., Kim B. (2025). Targeting natural antioxidant polyphenols to protect neuroinflammation and neurodegenerative diseases: A comprehensive review. Front. Pharmacol..

[236] Karakis I. (2025). Is There a Link Between Epilepsy and Intestinal Microbiota: What is Your gut Feeling?. Epilepsy Curr..

[237] Li J., Long Q., Ding H., Wang Y., Luo D., Li Z., Zhang W. (2024). Progress in the Treatment of Central Nervous System Diseases Based on Nanosized Traditional Chinese Medicine. Adv. Sci..

[238] Domínguez-López I., López-Yerena A., Vallverdú-Queralt A., Pallàs M., Lamuela-Raventós R.M., Pérez M. (2025). From the gut to the brain: The long journey of phenolic compounds with neurocognitive effects. Nutr. Rev..

[239] Kim J.-E., Cho K.-O. (2019). Functional Nutrients for Epilepsy. Nutrients.

[240] Wang B.H., Hou Q., Lu Y.Q., Jia M.M., Qiu T., Wang X.H., Zhang Z.X., Jiang Y. (2018). Ketogenic diet attenuates neuronal injury via autophagy and mitochondrial pathways in pentylenetetrazol-kindled seizures. Brain Res..

[241] Cryan J.F., O’Riordan K.J., Sandhu K., Peterson V., Dinan T.G. (2020). The gut microbiome in neurological disorders. Lancet Neurol..

[242] Vendrik K.E.W., Ooijevaar R.E., de Jong P.R.C., Laman J.D., van Oosten B.W., van Hilten J.J., Ducarmon Q.R., Keller J.J., Kuijper E.J., Contarino M.F. (2020). Fecal Microbiota Transplantation in Neurological Disorders. Front. Cell Infect. Microbiol..

[243] He Z., Cui B.T., Zhang T., Li P., Long C.Y., Ji G.Z., Zhang F.M. (2017). Fecal microbiota transplantation cured epilepsy in a case with Crohn’s disease: The first report. World J. Gastroenterol..

[244] Citraro R., Lembo F., De Caro C., Tallarico M., Coretti L., Iannone L.F., Leo A., Palumbo D., Cuomo M., Buommino E. (2021). First evidence of altered microbiota and intestinal damage and their link to absence epilepsy in a genetic animal model, the WAG/Rij rat. Epilepsia.

[245] Medel-Matus J.S., Shin D., Dorfman E., Sankar R., Mazarati A. (2018). Facilitation of kindling epileptogenesis by chronic stress may be mediated by intestinal microbiome. Epilepsia Open.

[246] Mishra P., Singh S.C., Ramadass B. (2024). Drug resistant epilepsy and ketogenic diet: A narrative review of mechanisms of action. World Neurosurg. X.

[247] Macrì R., Mollace R., Serra M., Scarano F., Ritorto G., Ussia S., Cardamone A., Coppoletta A.R., Carresi C., Gliozzi M. (2024). Nutritional and Nutraceutical Support to the Failing Myocardium: A Possible Way of Potentiating the Current Treatment of Heart Failure. Int. J. Mol. Sci..

[248] Serra M., Macrì R., Bonacci S., Ritorto G., Ussia S., Nucera S., Caminiti R., Ruga S., Altomare C., Tucci L. (2025). The second life of Citrus bergamia: Bioavailability analysis of a new formulation using waste-based microencapsulation as a valuable source of bioactive compounds. Pharmacol. Rep..

[249] Mollace R., Macrì R., Nicita M., Musolino V., Gliozzi M., Carresi C., Bava I., Maiuolo J., Tavernese A., Cardamone A. (2023). Bergamot Polyphenolic Extract Combined with Albedo and Pulp Fibres Counteracts Changes in Gut Microbiota Associated with High-Fat Diet: Implications for Lipoprotein Size Re-Arrangement. Int. J. Mol. Sci..

[250] Abbasi H., Khoshdooz S., Abbasi M.M., Eslamian G. (2025). Dietary Antioxidant Quality Score and Epilepsy Odds in the US Adults: A Cross-Sectional NHANES Study. Brain Behav..

[251] Zhong H., Jiang J., Hussain M., Zhang H., Chen L., Guan R. (2025). The Encapsulation Strategies for Targeted Delivery of Probiotics in Preventing and Treating Colorectal Cancer: A Review. Adv. Sci..

[252] Mejía-Granados D.M., Villasana-Salazar B., Lozano-García L., Cavalheiro E.A., Striano P. (2021). Gut-microbiota-directed strategies to treat epilepsy: Clinical and experimental evidence. Seizure.

[253] Weinstein N., Garten B., Vainer J., Minaya D., Czaja K. (2020). Managing the Microbiome: How the Gut Influences Development and Disease. Nutrients.

[254] Iannone L.F., Gómez-Eguílaz M., Citraro R., Russo E. (2020). The potential role of interventions impacting on gut-microbiota in epilepsy. Expert. Rev. Clin. Pharmacol..

[255] Ogunsuyi O.B. (2024). Functional Foods and Nutraceuticals in the Management of Epilepsy. Curr. Funct. Foods.

[256] He L.Y., Hu M.B., Li R.L., Zhao R., Fan L.H., He L., Lu F., Ye X., Huang Y.L., Wu C.J. (2021). Natural Medicines for the Treatment of Epilepsy: Bioactive Components, Pharmacology and Mechanism. Front. Pharmacol..

